# Cell-autonomous immune dysfunction driven by disrupted autophagy in *C9orf72*-ALS iPSC-derived microglia contributes to neurodegeneration

**DOI:** 10.1126/sciadv.abq0651

**Published:** 2023-04-21

**Authors:** Poulomi Banerjee, Arpan R. Mehta, Raja S. Nirujogi, James Cooper, Owen G. James, Jyoti Nanda, James Longden, Karen Burr, Karina McDade, Andrea Salzinger, Evdokia Paza, Judith Newton, David Story, Suvankar Pal, Colin Smith, Dario R. Alessi, Bhuvaneish T. Selvaraj, Josef Priller, Siddharthan Chandran

**Affiliations:** ^1^ UK Dementia Research Institute at University of Edinburgh, University of Edinburgh, Edinburgh bioQuarter, Chancellor’s Building, 49 Little France Crescent, Edinburgh EH16 4SB, UK.; ^2^ Centre for Clinical Brain Sciences, University of Edinburgh, Edinburgh EH16 4SB, UK.; ^3^ Euan MacDonald Centre for MND Research, University of Edinburgh, Edinburgh EH16 4SB, UK.; ^4^ Anne Rowling Regenerative Neurology Clinic, University of Edinburgh, Edinburgh EH16 4SB, UK.; ^5^ Medical Research Council (MRC) Protein Phosphorylation and Ubiquitylation Unit, School of Life Sciences, University of Dundee, Dow Street, Dundee DD1 5EH, UK.; ^6^ Edinburgh Brain Bank, Academic Department of Neuropathology, University of Edinburgh, Edinburgh, UK.; ^7^ Edinburgh Pathology, University of Edinburgh, Edinburgh, UK.; ^8^ Department of Psychiatry and Psychotherapy; School of Medicine, Technical University of Munich, Ismaninger Str. 22, 81675 Munich, Germany.; ^9^ Neuropsychiatry, Charité–Universitätsmedizin Berlin and DZNE, Charitéplatz 1, 10117 Berlin, Germany.

## Abstract

Although microglial activation is widely found in amyotrophic lateral sclerosis (ALS) and frontotemporal dementia (FTD), the underlying mechanism(s) are poorly understood. Here, using human-induced pluripotent stem cell–derived microglia-like cells (hiPSC-MG) harboring the most common ALS/FTD mutation (*C9orf72*, mC9-MG), gene-corrected isogenic controls (isoC9-MG), and *C9orf72* knockout hiPSC-MG (C9KO-MG), we show that reduced C9ORF72 protein is associated with impaired phagocytosis and an exaggerated immune response upon stimulation with lipopolysaccharide. Analysis of the C9ORF72 interactome revealed that C9ORF72 interacts with regulators of autophagy and functional studies showed impaired initiation of autophagy in mC9-MG and C9KO-MG. Coculture studies with motor neurons (MNs) demonstrated that the autophagy deficit in mC9-MG drives increased vulnerability of mC9-MNs to excitotoxic stimulus. Pharmacological activation of autophagy ameliorated both cell-autonomous functional deficits in hiPSC-MG and MN death in MG-MN coculture. Together, these findings reveal an important role for C9ORF72 in regulating immune homeostasis and identify dysregulation in myeloid cells as a contributor to neurodegeneration in ALS/FTD.

## Teaser

Disrupted autophagy led immune activation in microglia contributes to enhanced motor neuronal death in C9orf72-ALS.

## INTRODUCTION

Amyotrophic lateral sclerosis (ALS) is a devastating and incurable neurodegenerative disease characterized by selective loss of motor neurons (MNs) (*1*). Although the etiology of ALS is largely unknown, the finding that familial ALS (fALS) is clinically indistinguishable from sporadic ALS and shares a common pathology of TAR DNA-binding protein 43 (TDP-43) proteinopathy supports the study of monogenetic causes to better understand the unifying pathogenic mechanisms to develop effective treatments (*2*). For instance, a hexanucleotide repeat expansion in the intronic region of *C9orf72* is the most common known cause of fALS and frontotemporal dementia (FTD) (*3*). Proposed causative mechanisms underlying the *C9orf72* mutation are loss of function of C9ORF72 protein and/or gain of toxicity through RNA foci and dipeptide repeats (*4*).

Accumulating experimental and pathological evidence reveals that ALS is a multicellular disorder with involvement of not only MNs, but also other cell types, noting also that C9ORF72 is most highly expressed in myeloid cells (*5*–*7*). Microglia (MG), the resident immune cells of the CNS, are implicated in ALS, with evidence from autopsy *C9orf72*-ALS patient samples, suggesting chronic activation and dysregulation of the innate immune response (*8*, *9*). MG can be activated by a diverse range of stimuli, including, for example, neuronal damage signals released following excitotoxicity-mediated motor neuronal injury, a key proposed causal pathway in ALS (*10*–*12*). The finding of the immune regulatory function of C9ORF72 through suppression of stimulator of interferon genes (STING) activity opens the possibility that reduced elimination of immune responders, such as STING, in C9ORF72-deficient conditions, might be a driver of a dysregulated immune state in ALS (*13*, *14*).

Autophagy plays an important role in regulating inflammation and can be induced upon stimulation of Toll-like receptors (TLRs) (*15*). Immune function is modulated through selective degradation and/or disassembly of key inflammatory mediators, such as IκB kinase complex (IKKs)–activator for nuclear factor κB (NF-κB) pathway, nucleotide-binding domain, leucine-rich–containing family, pyrin domain–containing-3 (NLRP3)-inflammasome complex, and STING (*16*, *17*). Furthermore, loss of autophagy-associated proteins, such as ATG7, ATG5, and BECLIN, in macrophages results in enhanced production of proinflammatory cytokines in the presence of inflammasome activators, such as lipopolysaccharide (LPS) and nigericin (*18*, *19*). Impaired autophagy in myeloid cells has been reported to correlate with increased inflammation and exacerbation of neuronal damage in experimental models of neurodegeneration, including mice injected with β-amyloid and mice expressing human α-synuclein (*20*–*22*).

Against this background, using patient-derived MG-like cells from human induced pluripotent stem cells (hiPSCs) and macrophages, we examined the cell-autonomous and noncell-autonomous consequences of the *C9orf72* mutation.

## RESULTS

### mC9-MG exhibit phagocytic impairments and a hyper-active phenotype following LPS stimulation

**Fig. 1.**
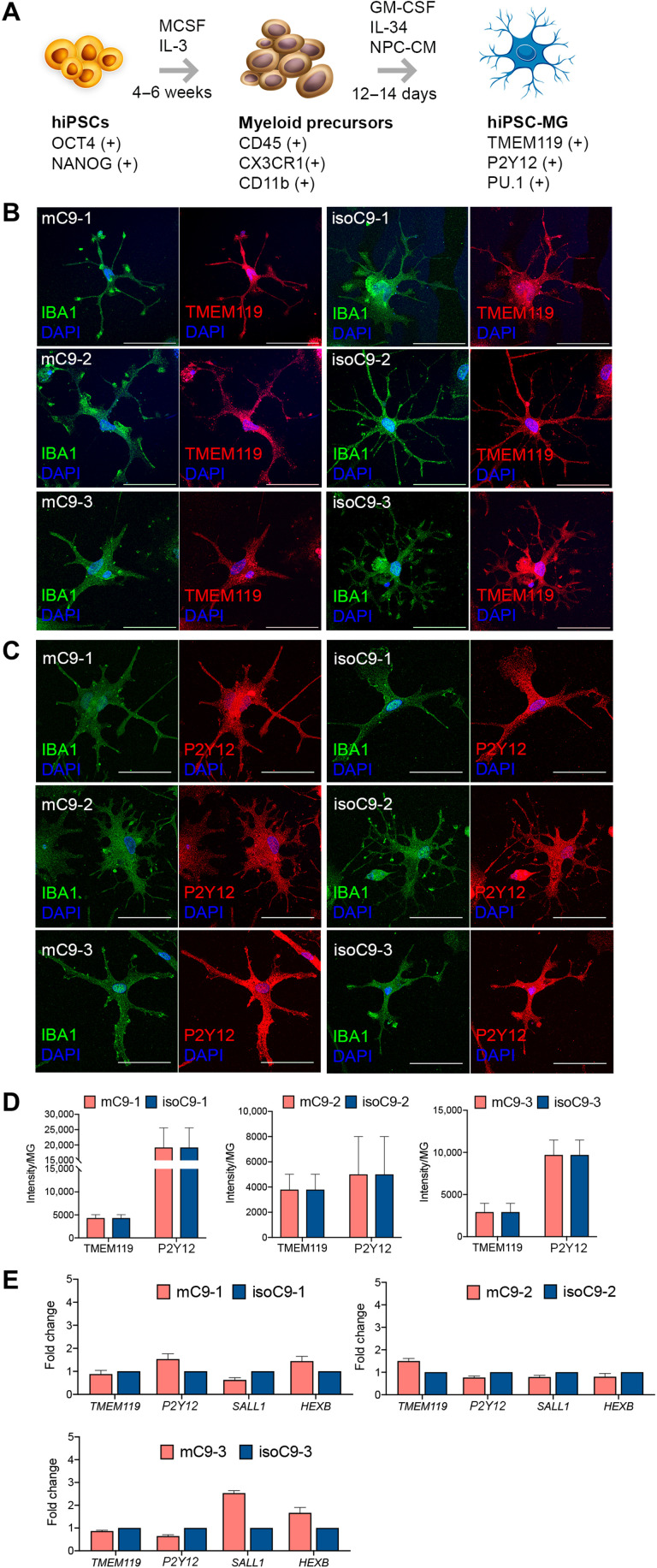
Generation and characterization of microglia-like cells (hiPSC-MG) from *C9orf72* mutant and isogenic iPSC lines. (**A**) Schematic depicting stages, timeline, and key markers for the differentiation of microglia-like cells (hiPSC-MG) from hiPSCs. (**B** and **C**) Representative immunofluorescence images of mC9-MG and isoC9-MG demonstrating comparable staining of microglial markers: TMEM119 (red) IBA-1 (green) and P2Y12 (red) IBA1 (green) generated from three pairs of *C9orf72* mutant and isogenic iPSC lines. Scale bars, 50 μm. DAPI, 4′,6-diamidino-2-phenylindole. (**D**) Bar graph representing the quantification of fluorescence intensity per hiPSC-MG for TMEM119 and P2Y12 across three pairs of mC9-MG and isoC9-MG. (**E**) Bar graphs representing the expression of MG signature genes (*TMEM119*, *P2Y12*, *SALL1*, and *HEXB*) in mC9-MG and isoC9-MG across three pairs. Data are represented as means ± SD; *N* = 3, where *N* represents the number of times experiments were performed using cells generated from independent iPSC differentiations.

**Fig. 2.**
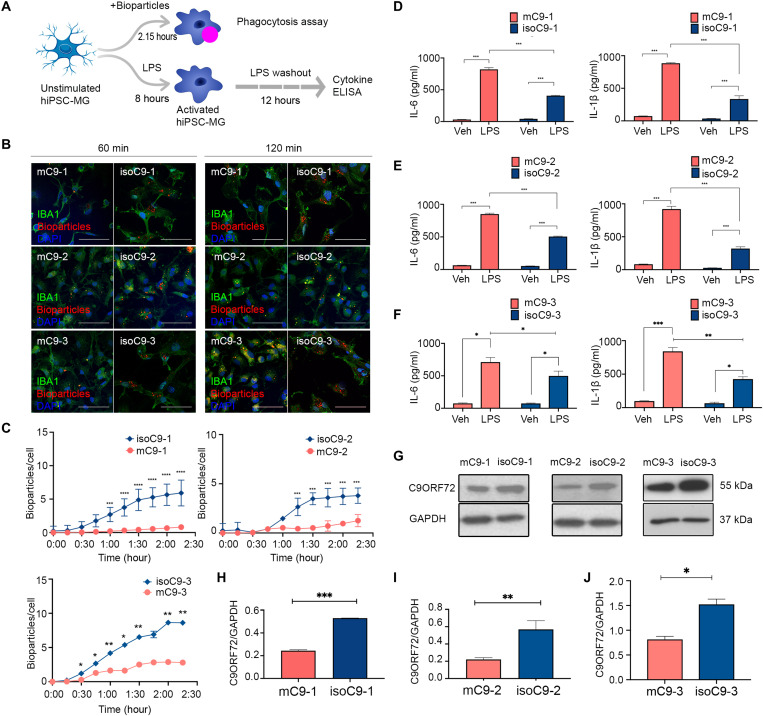
mC9-MGs display impaired phagocytosis and heightened immune response following LPS stimulation. (**A**) Schematic overview of the experimental paradigm for the assessment of microglial function—phagocytosis and immune response following LPS stimulation. ELISA, enzyme-linked immunosorbent assay. (**B**) Representative images of immunofluorescence staining showing phagocytosis assay performed with pH-sensitive zymosan bioparticles for three pairs of mC9-MG and isoC9-MG at 60 and 120 min [IBA-1 (green), zymosan bioparticles (red)]. Scale bars, 50 μm. (**C**) Graphs showing real-time imaging of zymosan bioparticle uptake at 15-min intervals, demonstrating a phagocytic deficit in mC9-MG when compared to isoC9-MGs across three pairs. Statistical analysis was performed across mC9-MG and isoC9-MG using two-way analysis of variance (ANOVA) and Tukey’s multiple comparisons test; data are represented as means ± SEM, *N* = 3 (*P < 0.05, **P < 0.01, ***P < 0.001, and ****P < 0.0001). (**D** to **F**) Graphs showing increased production of IL-6 and IL-1β in mC9-MG (red) compared to isoC9-MG (blue) following LPS stimulation, cells were treated with either LPS or 1× phosphate-buffered saline (PBS) [vehicle control (veh)]. Statistical analysis was performed using two-way ANOVA and Tukey’s multiple comparisons test, data are represented as means ± SD; *N* = 3 (*P < 0.05;, **P < 0.01, and ***P < 0.001). (**G** to **J**) Immunoblot with respective densitometric analysis showing the reduced abundance of C9ORF72 protein (~55 kDa) across three lines of mC9-MG (red) when compared to their respective isogenics (blue). Data are represented as means ± SD; *N* = 3. Statistical analysis was performed using Student’s *t* test (*P < 0.05, **P < 0.01, and ***P < 0.001). *N* across all experiments represents the number of times experiments were performed using cells generated from independent iPSC differentiations. GAPDH, glyceraldehyde-3-phosphate dehydrogenase.

To understand the cell-autonomous consequence of the *C9orf72* mutation on human MG, we first generated monocultures of hiPSC-MG, using established protocols (*23*, *24*), from three pairs of *C9orf72*-ALS patient iPSC lines (mC9-1, mC9-2, and mC9-3-MG) and their paired gene-edited isogenic controls (isoC9-1, isoC9-2, and isoC9-3-MG) (Fig. 1A and table S1) (*11*). Briefly, embryoid bodies made from iPSCs were treated with macrophage colony-stimulating factor (M-CSF) and interleukin-3 (IL-3) to generate myeloid precursors expressing CD11b, CX3CR1, and CD45 (fig. S1, A to D). These myeloid precursors were then exposed to IL-34, granulocyte macrophage colony-stimulating factor (GM-CSF), and neural precursor cell-conditioned media (NPC-CM) to generate hiPSC-MGs (fig. S1E). mC9-MG and isoC9-MG expressed equivalent levels of microglial markers both at mRNA (*TMEM119*, *P2RY12*, *SALL1*, and *HEXB*) and protein (TMEM119, P2Y12, and PU.1) levels, and no significant difference was observed in morphometric parameters (such as area, branching, and major axis of cell) between mC9-MG and isoC9-MGs (Fig. 1, B to E, and fig. S2, A to C). To assess the impact of *C9orf72* mutation on microglial function, we measured the phagocytic and cytokine production response following LPS stimulation (Fig. 2A). Phagocytosis assay revealed significantly fewer internalized pH-sensitive zymosan bioparticles (Fig. 2B), resulting from a reduction in the rate of internalization at every time point assessed in mC9-MG compared to isoC9-MG (Fig. 2C and fig. S3). We next quantified the microglial production of proinflammatory cytokines (IL-6 and IL-1β) in response to LPS stimulation. Although the levels of IL-6 and IL-1β were comparable under basal condition across m*C9* and iso*C9*-MGs, significantly elevated levels of IL-6 and IL-β were evident in mC9-MG upon treatment with LPS compared with isoC9-MG (Fig. 2, D to F). These findings show that the *C9orf72* mutation leads to impaired microglial phagocytosis and a hyper-activated immune phenotype following LPS stimulation.

### C9ORF72 interactome reveals association with regulators of autophagy in hiPSC-MG

**Fig. 3.**
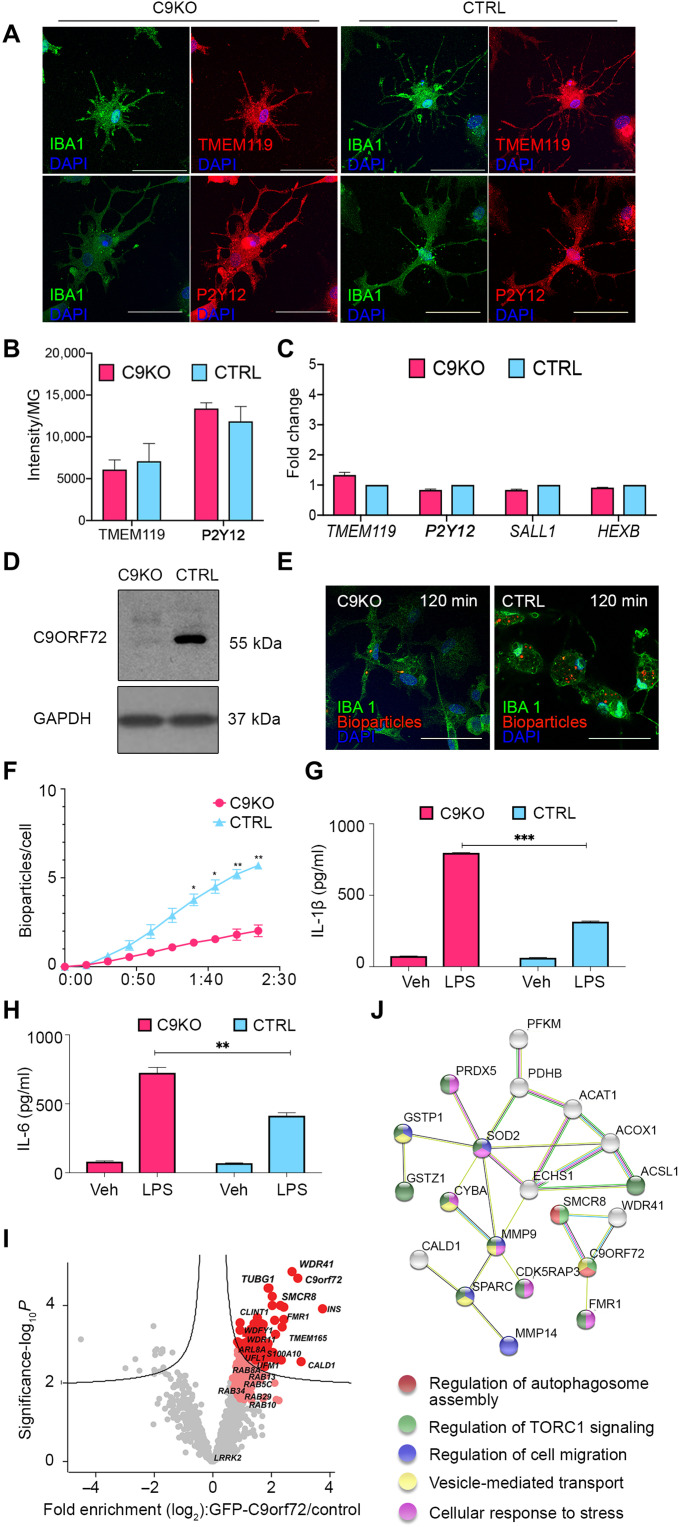
C9KO-MG phenocopies functional deficits found in mC9-MG and mass spectrometric analysis of C9ORF72 interactome revealed its association with regulators of autophagy. (**A**) Represents immune staining of C9KO-MG and CTRL-MG from the same genetic background confirming positive staining for microglial markers such as TMEM119 (red) and P2Y12 (red) along with IBA1 (green). Scale bars, 50 μm. (**B**) Bar graph representing equivalent fluorescence intensity per hiPSC-MG for TMEM119 and P2Y12 across C9KO-MG and CTRL-MG. (**C**) Bar graph representing the comparable expression of MG signature genes (*TMEM119*, *P2Y12*, *SALL1*, and *HEXB*) in C9KO-MG and CTRL-MG. Data are represented as means ± SD; *N* = 3. (**D**) Immunoblot validating loss of C9ORF72 protein (55 kDa) in C9KO-MG. (**E**) Representative images of immunofluorescence staining showing phagocytosis assay performed with pH-sensitive zymosan bioparticles for C9KO-MG and CTRL-MG at 120 min [IBA-1 (green), zymosan bioparticles (red)]. Scale bars, 50 μm. (**F**) Graph showing real-time imaging of zymosan bioparticle uptake at 15-min intervals, demonstrating a phagocytic deficit in C9KO-MG when compared to control (CTRL-MG). Statistical analysis was performed using two-way ANOVA and Tukey’s multiple comparisons test. Data are represented as means ± SEM (*P ≤ 0.05 and **P ≤ 0.01); *N* = 3. (**G** and **H**) Graphs demonstrating the increased production of IL-1β and IL-6 for C9KO-MGs following LPS stimulation; cells were treated with either LPS or 1× PBS [vehicle control (veh)]. Statistical analysis was performed using two-way ANOVA and Tukey’s multiple comparisons test. Data are represented as means ± SD; *N* = 3 (**P ≤ 0.01 and ***P ≤ 0.001). (G) Volcano plot demonstrating enrichment of C9ORF72 interactors at 1% false discovery rate (FDR) (dark red) and 5% FDR (light red). *n* = 4. (H) STRING network analysis of the C9ORF72 interactors (1% FDR); nodes represent the functional enrichment of the network. *N* for all experiments represents the number of times experiments were performed using cells generated from independent iPSC differentiations.

C9ORF72 protein is highly expressed in myeloid cells (*5*–*7*), a finding that we also confirmed by the quantitative immunoblot of control hiPSC-MG, hiPSC-neurons, and hiPSC-astrocytes (fig. S4). Immunoblot of mC9-MG compared to isoC9-MG across three pairs revealed significantly reduced abundance of C9ORF72 protein (Fig. 2, G to J), a finding in line with previous reports (*25*). To further investigate the impact of reduced C9ORF72 protein on microglial function, we generated hiPSC-MG from a *C9orf72* null iPSC line. The *C9orf72* null iPSC line was generated from a control iPSC line using CRISPR-Cas9 genome-editing technology (fig. S5A). C9KO-MGs and CTRL-MGs expressed equivalent levels of MG markers—TMEM119, P2Y12, PU.1, and MG signature genes (*TMEM119*, *P2RY12*, *SALL1*, and *HEXB*) (Fig. 3, A to C, and fig. S5B). Immunoblot of C9KO-MG confirmed the absence of C9ORF72 protein (Fig. 3D) compared to the parental control iPSC-derived MG (CTRL-MG). C9KO-MG also exhibited reduced phagocytosis (Fig. 3, E and F) and increased production of IL-1β and IL-6 following LPS stimulation (Fig. 3, G and H), thereby phenocopying the microglial functional deficits found in mC9-MG. These findings suggest that the reduced abundance of C9ORF72 protein contributes to the functional deficits seen in mC9-MG. To better understand the function and the pathways that are disrupted due to loss of C9ORF72 in hiPSC-MG, we next evaluated the interactome of C9ORF72 in CTRL-MG. A C9ORF72 reporter iPSC line was generated by introducing an enhanced green fluorescent protein (EGFP) tag at the N terminus of the wild-type C9ORF72 (preserving its endogenous promoter) to perform GFP-Trap immunoprecipitation (fig. S6A). The EGFP-C9 line was differentiated into EGFP C9-MG (fig. S6C), and immunoblot with GFP antibody and C9ORF72 antibody confirmed the presence of EGFP-C9ORF72 fusion protein (fig. S6, B and D). Following immunoprecipitation using GFP-Trap beads, the cell lysate was subjected to an unsupervised proteomic screen to identify the interaction partners of EGFP-C9ORF72 by tandem mass spectrometry. Applying stringent pretest criteria of >2.0-fold enrichment and 1% false discovery rate (FDR), 254 potential interactor proteins, such as FMR1, MMP9, and UFL1, were identified (Fig. 3I and table S2). STRING network analysis was next performed on the top 50 proteins (GFP versus control, fold enrichment ≥3.0). The five most significant Gene Ontology processes (*P* > 0.05) identified were (i) regulation of TORC1 signaling, (ii) regulation of autophagosome assembly, (iii) vesicle-mediated transport, (iv) regulation of cell migration, and (v) cellular response to stress (Fig. 3J). Among these, autophagy-associated proteins, such as SMCR8 and WDR41, were enriched more than fivefold; these were further validated using Western blotting (fig. S6D). Last, enrichment for multiple autophagy-associated RAB proteins (RAB5, RAB8A, RAB39Ab, RAB13, RAB 34, RAB21, and RAB10) was observed in the C9ORF72 interactome, in line with previous findings (*26*). Together, these findings suggest an important role of C9ORF72 in the regulation of autophagy in human MG.

### *C9orf72* loss of function disrupts autophagy initiation in mC9-MG

**Fig. 4.**
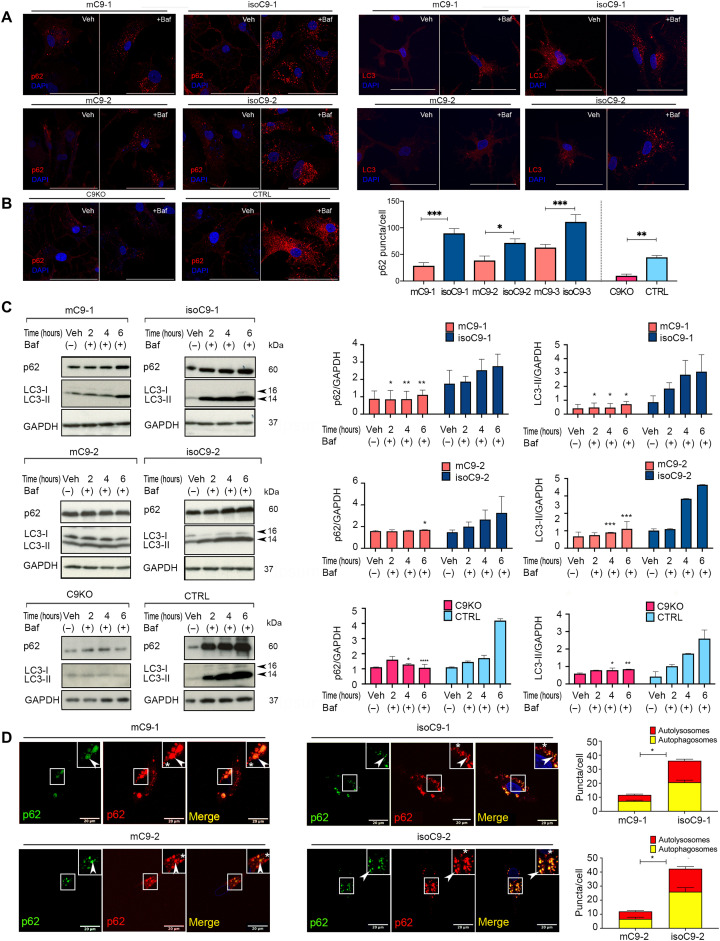
mC9-MG demonstrate a deficit in the initiation of autophagy. (**A**) Representative images of immunofluorescence staining of p62- (left) and LC3- (right)–positive puncta after 6 hours of dimethyl sulfoxide (DMSO) (vehicle control) and bafilomycin (Baf) treatment. (**B**) Representative images of immunofluorescence staining showing p62 + ve puncta at 6 hours after bafilomycin treatment in C9KO-MG when compared to CTRL-MG. Scale bars, 50 μm. Right: Bar graph demonstrating a reduced number of p62 + ve puncta per cell in mC9-MG and C9KO-MG after 6 hours of bafilomycin treatment. Data are represented as means ± SD; *N* = 3 (*n* = 20 cells per genotype). Statistical analysis was performed using two-way ANOVA and Tukey’s multiple comparisons test. **P < 0.05, **P < 0.01, and ***P < 0.001. (**C**) Representative immunoblots (left) showing the reduced turnover of p62 (~60 kDa) and LC3-II (~15 kDa) in two pairs of mC9-MG and C9KO-MG compared to their respective isoC9-MG and CTRL-MG in the presence of bafilomycin at 2, 4, and 6 hours. Bar graphs (right) represent the densitometric quantification of p62 and LC3-II normalized to loading control glyceraldehyde-3-phosphate dehydrogenase(GAPDH). Data are represented as means ± SD; *N* = 3. Statistical analysis was performed across mC9-MG, isoC9-MG and C9KO-MG, and CTRL-MG at 2, 4, and 6 hours using two-way ANOVA and Sidak’s multiple comparisons test. *P < 0.05, **P < 0.01, ***P < 0.001, and ****P < 0.0001. (**D**) Representative images from live imaging of mcherry-EGFP-p62 dual-reporter probe transduced in two pairs of mC9-MG and isoC9-MG; white arrowhead in the inset represents autophagosomes (GFP^+^, mCherry^+^) and white asterisk represents autolysosomes (GFP^−^mCherry^+^). Stacked bar graphs (right) demonstrating the quantification of autophagosomes and autolysosomes. Statistical analysis was performed using two-way ANOVA and Tukey’s multiple comparisons test for number of autophagosomes across mC9-MG and isoC9-MG. Data are represented as means ± SD; *N* = 3 [*n*(mC9-1 = 20 cells, mC9-2 = 18 cells, isoC9-1 = 20 cells, isoC9-2 = 20 cells)]; *P < 0.05. *N* represents the number of times experiments were performed using cells generated from independent iPSC differentiations.

Recognizing that the C9ORF72 interactors, SMCR8, WDR41, and RAB5, are integral to the autophagy initiation machinery (*27*, *28*), we first investigated basal autophagy in mC9-MG. We measured the formation of autophagosomes in the presence of 100 nM bafilomycin (preventing the fusion of autophagosomes and lysosomes) by quantitative immunostaining with the autophagosome membrane proteins, LC3 and p62. Upon bafilomycin treatment, we observed significantly fewer LC3 and p62 puncta in the mC9-MG when compared to isoC9-MG (Fig. 4, A and B, and figs. S7A and S8, A to D). C9KO-MGs, compared to CTRL-MG, also displayed a significantly reduced number of p62 puncta after bafilomycin treatment (Fig. 4B), and there was no significant difference in the number of p62 puncta across mC9-MG, isoC9-MG, C9KO-MG, and CTRL-MG in the vehicle-treated condition (fig. S8E). Furthermore, the autophagy deficit was also confirmed by immunoblot which demonstrated reduced levels of p62 and LC3 at 2, 4, and 6 hours in the presence of bafilomycin in mC9-MGs and C9KO-MG compared to their isogenic controls and CTRL-MG, respectively (Fig. 4C and fig. S9). To explore whether this autophagic deficit reflected a deficit in the induction of autophagy, we next measured autophagy flux using the lentiviral overexpression of a tandem dual-fluorescent reporter (mCherry-GFP-p62) (*29*). This pH-sensitive tool enables the assessment of autophagosomes that are GFP^+^ and mCherry^+^ (yellow puncta) versus autolysosomes that are GFP^−^ and mCherry^+^ (red puncta). We observed significantly fewer autophagosomes in mC9-MG and C9KO-MG compared to isogenic controls and CTRL-MG (Fig. 4D and fig. S7C). Furthermore, we found that the ratio of autolysosomes to autophagosomes was not altered in mC9-MG (Fig. 4D and fig. S7C), a finding consistent with the autophagic deficit in mC9-MG being driven by an impairment in the induction of autophagy. This deficit in autophagy also became evident during phagocytosis, where reduced numbers of LC3 puncta were observed during the internalization of zymosan beads in mC9-MG compared to their isoC9-MG (fig. S10). Thus, we show deficits in autophagy initiation in mC9-MG and C9KO-MG.

### Disrupted autophagy leads to sustained activation of NLRP3-inflammasome and NF-κB signaling in mC9-MG

**Fig. 5.**
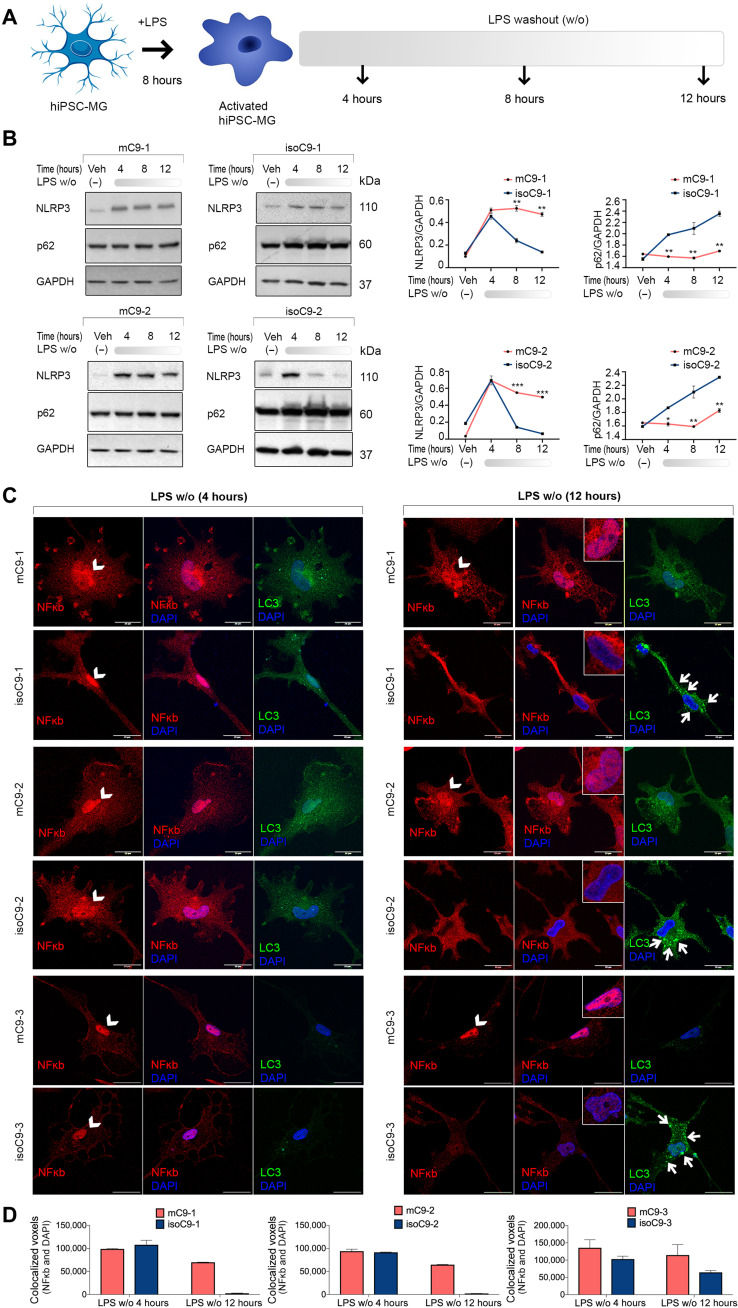
Disrupted autophagy in mC9-MG results in inadequate attenuation of inflammasome activation and NF-κB signaling following LPS stimulation. (**A**) Experimental paradigm for assessing NLRP3 inflammasome dynamics and NF-κB signaling following LPS stimulation. mC9-MG and isoC9-MG were treated with LPS (100 ng/ml) for 8 hours, and following LPS removal, whole-cell lysates were harvested at 4, 8, and 12 hours; cells were treated with either LPS or 1× PBS [vehicle control (veh)]. (**B**) Immunoblots showing immunoreactivity for NLRP3 (~110 kDa), p62 (~60 kDa), and corresponding line graphs depicting the densitometric quantification of NLRP3 and p62, normalized to GAPDH at 4, 8, and 12 hours demonstrating a sustained activation of NLRP3 and reduced p62 in mC9-MG. Data are represented as means ± SD for three independent experiments; statistical analysis across mC9-MG and isoC9-MG at 4, 8, and 12 hours was performed using two-way ANOVA and Sidak’s multiple comparisons test. *P < 0.05, **P < 0.01, and ***P < 0.001. (**C**) Representative images of immunofluorescence staining for NF-κB and LC3 demonstrating the dynamics of NF-κB signaling and autophagy activation respectively at 4 and 12 hours after LPS washout for three pairs of mC9-MG and isoC9-MG. The nuclear localization of NF-κB is indicative of activated NF-κB signaling, and the appearance of LC3 puncta indicates autophagy induction. The nuclear region is highlighted using white arrowheads, and the presence of LC3 puncta are indicated using small white arrows at the 12-hour time point. Scale bars, 20 μm. (**D**) Graphs show the quantification of colocalized voxels of NF-κB and DAPI demonstrating the sustained activation of NF-κB signaling in mC9-MG. Data are represented as means ± SD, *N* = 3. Data represent *n* = 20 cells across all genotypes. *N* represents the number of times experiments were performed using cells generated from independent iPSC differentiations.

Since autophagy regulates the attenuation of proinflammatory signaling (*30*), we next investigated the impact of the *C9orf72* mutation on the NLRP3 inflammasome and NF-κB pathway in mC9-MG. We treated mC9-MG and isoC9-MG in a time-limited exposure (8 hours) with LPS to stimulate an immune response. Following this, we removed LPS and monitored the postwashout time course through the collection of cellular lysates at 4, 8, and 12 hours (Fig. 5A). Following the removal of LPS stimulation, we observed a comparable increase in the levels of NLRP3 in mC9-MG, isoC9-MG, and CTRL-MG during the first 4 hours after LPS washout, indicating a comparable inflammatory response to LPS stimulation (Fig. 5B and fig. S11, A and B). However, in CTRL-MG and isoC9-MG, levels of NLRP3 returned to near-basal levels between 8 and 12 hours (Fig. 5B and fig. S11, A and B). This reduction in NLRP3 protein was accompanied by a reciprocal increase in p62 levels, marking the activation of autophagy, over the same time interval (Fig. 5B and fig. S11, A and C) in CTRL-MG and iso*C9*-MG. In contrast, in mC9-MG, there was no significant reduction in the level of NLRP3, and p62 levels also remained significantly reduced at 4, 8, and 12 hours following LPS removal (Fig. 5B). Beside LPS stimulation (one-step stimulation model), we further tested the dynamics of NLRP3 and LC3 in mC9-MG and isoC9-MG using a two-step stimulation model using adenosine triphosphate (ATP) as a secondary stimulus. Both LPS-primed and LPS-untreated mC9-MG and isoC9-MG were stimulated with 1 mM ATP for 30 min, and NLRP3 intensity was first measured at 30 min and also following a washout interval of 12 hours (fig. S11F). Our findings revealed an elevated, although comparable, increase in NLRP3 for mC9-MG and isoC9-MG at 30 min after treatment with LPS + ATP compared to LPS alone (fig. S11, G, H, and J). However, in contrast to mC9-MG, a significant reduction in the level of NLRP3 accompanied by a concomitant increase of LC3 puncta (indicative of autophagy induction) was observed in isoC9-MG at 12 hours after LPS, ATP, and LPS + ATP treatment (fig. S11, I and K). Thus, we detected a sustained activation of NLRP3 signaling in mC9-MG in the absence of autophagy activation. Together, these findings across different models of stimulation are consistent with a mutation-dependent impairment in the autophagy initiation machinery, resulting in the sustained activation of NLRP3-inflammasome following LPS induction.

We next investigated NF-κB pathway dynamics, another inflammatory pathway reliant on autophagy for negative regulation (*31*). Four hours after LPS removal, in both mC9 and their isogenic pairs, we observed the nuclear localization of NF-κB (Fig. 5C), a finding that implies inflammation-induced activation of this pathway. Note that, under basal conditions, NF-κB is largely localized to the cytoplasm (fig. S12). This activated state, as evidenced by nuclear localization, exhibited a tendency to return to basal state (cytoplasmic localization) in iso*C9*-MG and CTRL-MG at 12 hours (Fig. 5C and fig. S11, D and E). In contrast, mC9-MG displayed the sustained nuclear localization of NF-κB at 12 hours after LPS washout (Fig. 5, C and D). Collectively, these data suggest that the *C9orf72* mutation leads to the sustained activation of both NLRP3 and NF-κB signaling in iPSC-MG following LPS stimulation.

### Pharmacological activation of autophagy ameliorates the impaired phagocytosis and hyper-active state of mC9-MG and C9KO-MG

**Fig. 6.**
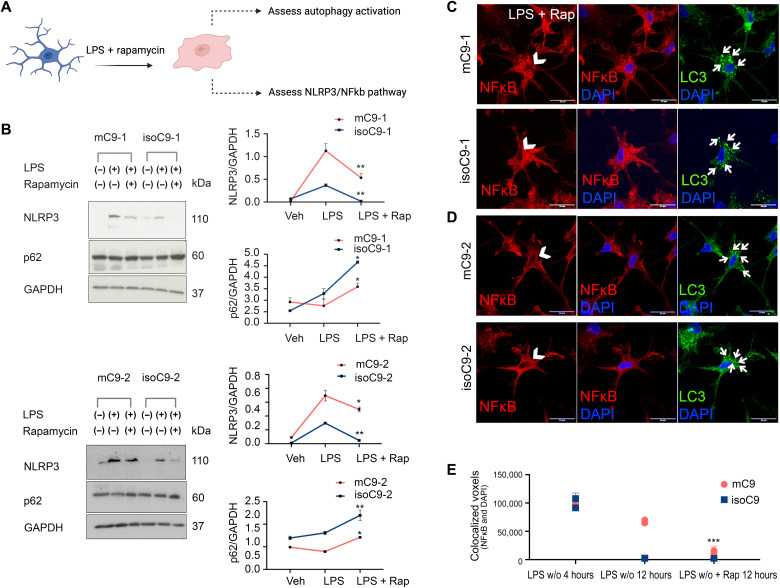
Pharmacological activation of autophagy with rapamycin leads to the attenuation of NLRP3 inflammasome and NF-κB signaling in mC9-MG following LPS stimulation. (**A**) Schematic showing the experimental setup wherein the cells were treated with rapamycin for 12 hours, during LPS washout, and were tested for activation of autophagy and state of NLRP3/NF-κB signaling. (**B**) Immunoblots and their quantification showing an increase in p62 level (~60 kDa) and a reduction in NLRP3 level (~110 kDa) across mC9-MG and isoC9-MG in the presence of rapamycin. The cells were either treated with vehicle (veh), LPS, or LPS + rapamycin (LPS + Rap). Statistical analysis was performed for LPS-primed mC9-MG and isoC9-MG across rapamycin-treated and rapamycin-untreated condition using two-way ANOVA and Sidak’s multiple comparisons test. *P < 0.05 and **P < 0.01. Data represent means ± SD across three independent experiments. (**C** and **D**) Representative images of immunofluorescence staining showing the reduction of nuclear localization of NF-κB in rapamycin-treated mC9-MG, demonstrating an attenuation of NF-κB signaling. The nuclear regions are indicated using white arrowheads and the concurrent increase in the appearance of LC3 puncta is highlighted using white arrows. Scale bars, 20 μm. (**E**) Quantification of the colocalized voxels of NF-κB and DAPI after rapamycin treatment. Data are represented as means ± SD; *n* = 20 cells across all genotypes. Statistical analysis was performed for mC9-MG and isoC9-MG across rapamycin-treated and -untreated condition in the presence of LPS at 12-hour time point using two-way ANOVA and Sidak’s multiple comparisons test. ***P < 0.001.

**Fig. 7.**
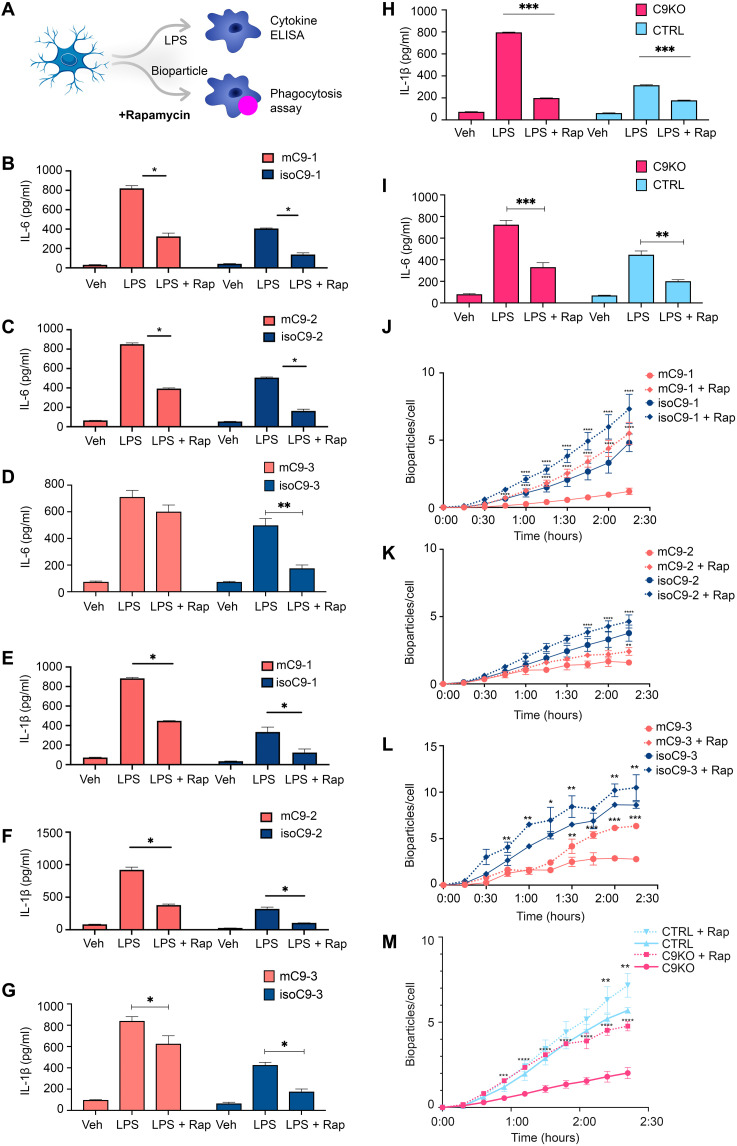
Pharmacological activation of autophagy with rapamycin ameliorates the sustained immune activation and phagocytic deficit in mC9-MG and C9KO-MG. (**A**) Schematic showing the experimental setup wherein the cells are treated with rapamycin for 12 hours and tested for cytokine release and phagocytosis of zymosan bioparticles. (**B** to **G**) Cytokine ELISA of IL-6 and IL-1β demonstrates suppression of the production of proinflammatory cytokines in mC9-MG following rapamycin treatment. Cells were treated with either vehicle (veh), LPS, or LPS + rapamycin (LPS + Rap). Data are represented as means ± SD; statistical analysis was performed using two-way ANOVA and Sidak’s multiple comparisons test (*P ≤ 0.05); *N* = 3. (**H** and **I**) IL-1β and IL-6 ELISA demonstrates the amelioration of immune response in C9KO-MGs mirroring mC9-MGs as a result of rapamycin treatment; cells were treated with either vehicle (veh), LPS, or LPS + rapamycin. Data are represented as means ± SD; statistical analysis was performed using two-way ANOVA and Sidak’s multiple comparisons test. (**P < 0.01 and ***P < 0.001); *N* = 3. (**J** to **M**) Graph showing real-time imaging of zymosan bioparticle uptake assay demonstrating rapamycin-mediated amelioration of the phagocytic deficit in mC9-MGs and C9KO-MG; statistical analysis was performed across rapamycin-treated and rapamycin-untreated condition for all genotypes using two-way ANOVA and Tukey’s multiple comparisons test (*P <0.05, **P < 0.01, ***P < 0.001, and ****P < 0.0001) and error bars represent ± SEM; *N* = 3. *N* represents the number of times experiments were performed using cells generated from independent differentiations from iPSCs.

To determine whether boosting autophagy could ameliorate both the phagocytic deficit and activated immune state of mC9-MG, we next treated mC9-MG with 10 μM rapamycin, a pharmacological activator of autophagy (Fig. 6A) (*32*). Rapamycin treatment resulted in a significant increase of p62-positive puncta in bafilomycin-treated mC9-MG (figs. S7B and S13, A and B) and C9 KO-MG (fig. S13, C and D). This finding was further validated by Western blot analysis, demonstrating a time-dependent increase in p62 level for mC9-MG, isoC9-MG, C9KO-MG, CTRL-MG, and LC3(II) levels for mC9-MG and isoC9-MG following rapamycin treatment in the presence of bafilomycin, reflective of autophagic induction (fig. S13, E to J). We next challenged mC9-MG, isoC9-MG, and CTRL-MG with LPS in the presence of rapamycin to investigate the impact on NLRP3 and p62 expression. Notably, we observed a significant reduction in NLRP3 immunoreactivity and a significant increase in p62 level at a steady-state following LPS stimulation in the mC9-MG, isoC9-MG, and CTRL-MG compared to mC9-MG that were not treated with rapamycin at 12 hours (Fig. 6B and fig. S14B). This rapamycin-induced amelioration of the hyper-activated state of mC9-MG was also found in the NF-κB signaling pathway, which demonstrated a reduction in the nuclear localization of NF-κB and an increase in the appearance of LC3 puncta, indicative of autophagy-mediated suppression of NF-κB signaling (Fig. 6, C to E, and fig. S14A). Next, we determined whether boosting autophagy in mC9-MG and C9KO-MG reverses the functional deficits of phagocytosis and increases cytokine production (Fig. 7A). Rapamycin treatment resulted in a significant reduction of the LPS-induced production of the proinflammatory cytokines IL-6 and IL-1β in mC9-MG, C9KO-MG, isoC9-MG, and CTRL-MG (Fig. 7, B to I) and also ameliorated the phagocytic deficit in mC9-MG and C9KO-MG (Fig. 7, J to M).

### Disrupted autophagy in mC9-MG contributes to enhanced motor neuronal death following excitotoxic insult

**Fig. 8.**
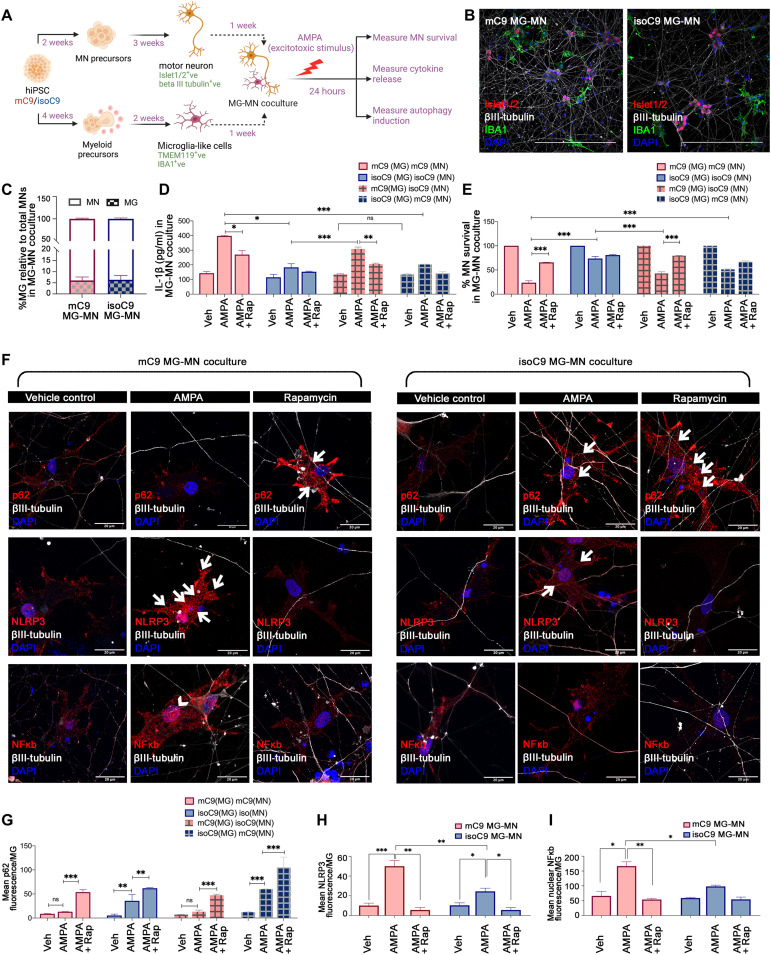
Disrupted autophagy in mC9-MG contributes to enhanced motor neuronal death following excitotoxic insult. (**A**) Schematic showing protocol for coculture of iPSC-derived MG and MNs (MG-MNs) to assess the impact of MG on MN survival, induction of autophagy in MG and cytokine release following excitotoxic stimulus (AMPA). (**B**) Representative immunofluorescence images of IBA1 (green) βIII-tubulin (gray) and Islet1/2 (red) in MG-MN coculture. Scale bars, 50 μm. (**C**) Graph representing percentage of MG relative to total MN in MG-MN coculture. Data are represented as means ± SD; *N* = 3. (**D**) Graph representing production of IL-1β in MG-MN cocultures in vehicle-treated condition, following AMPA challenge and in the presence of rapamycin. Data are represented as means ± SD; *N* = 3; *P < 0.05, **P < 0.01, and ***P < 0.001; ns, not significant. (**E**) Graphs showing percentage survival of MNs in MG-MN cocultures in vehicle-treated condition, following AMPA treatment and in the presence of rapamycin. Data are represented as means ± SD; *N* = 3. ***P < 0.001. (**F**) Representative images of fluorescence staining for microglial p62/NLRP3/NF-κB across vehicle-treated, AMPA-treated conditions and in the presence of rapamycin in MG-MN coculture. White arrows indicate the cytoplasmic localization of p62 and NLRP3 staining and arrowheads indicate the nuclear localization of NF-κB staining. Scale bars, 20 μm. (**G**) Graph representing the quantification of mean fluorescence intensity of p62 per microglial cell across vehicle-treated, AMPA-treated conditions and in the presence of rapamycin in MG-MN coculture. (**H** and **I**) Graph representing the quantification of the mean fluorescence intensity of NLRP3/NF-κB per microglial cell across vehicle-treated, AMPA-treated conditions and in the presence of rapamycin in MG-MN coculture. Twenty microglial cells from three biological replicates have been analyzed per condition against each genotype. Data are represented as means ± SD; *N* = 3. *P < 0.05, **P < 0.01, and ***P < 0.001; ns, not significant. *N* represents number of times experiments were performed using cells generated from independent iPSC differentiations.

To examine the functional impact of disrupted microglial autophagy on C9 mutant MNs (mC9-MNs), we undertook coculture studies of mC9-MG and mC9-MN (mC9 MG-MN), isoC9-MG and isoC9-MN (isoC9 MG-MN), and CTRL MN and CTRL MG (CTRL MG-MN) (Fig. 8, A and B, and fig. S15A). Spinal MNs were generated using an established protocol (*11*, *33*). We observed the comparable survival of MN across mC9 MG-MN, isoC9 MG-MN, and CTRL MG-MN under basal conditions (Fig. 8C and fig. S15E). We next treated these cocultures with AMPA to induce excitotoxicity (Fig. 8A and fig. S15B), as previously shown for isolated mC9-MN (fig. S15C) (*11*, *25*, *34*). Noting that neuronal excitotoxicity has been shown to induce microglial activation, we hypothesized that the mC9 MG-MN coculture would show increased MN death compared to MN cultures alone following 24-hour treatment with 100 μM AMPA (*12*). Using IL-1β as a marker of activated MG, we first demonstrated that AMPA does not elicit microglial activation in MG cultures alone (mC9, isoC9, and CTRL) (fig. S15F). However, we observed the significantly increased IL-1β expression of mC9-MG in the mC9MG-MN cocultures when compared to isoC9-MG in isoC9MG-MN and CTRL MG in CTRLMG-MN cocultures (Fig. 8D and fig. S15G). To further confirm that increased activation of mC9-MG in the coculture is a cell-autonomous dysregulation, we cocultured mC9-MG with isoC9-MN and isoC9-MG with mC9-MN. We again noted significantly increased IL-1β expression in mC9-MG:isoC9-MN coculture following AMPA stimulation when compared to isoC9-MG cultured with isoC9-MN (Fig. 8D). There was no significant change in the level of IL-1β across isoC9-MG:mC9-MN and isoC9-MG:isoC9-MN, thereby revealing that an activated state of mC9-MG is a consistent phenotype following AMPA stimulation, irrespective of the genotype of MN that they are cocultured with (Fig. 8D). Next, we assessed the survival of MN (mC9, isoC9, and CTRL) in isolation and when cocultured with matched/mismatched genotype MG upon AMPA stimulation. As previously reported, isolated mC9MN cultures revealed significantly reduced MN survival compared to isoC9MN (mC9-57 ± 4.72% versus isoC9-71 ± 1.73%) and CTRL-MN following AMPA stimulation (fig. S15C). AMPA treatment of mC9 MG-MN compared to isoC9 MG-MN and CTRL MG-MN cocultures revealed a significantly reduced mC9 MN survival compared to isoC9-MN (mC9 23 ± 3.46% versus isoC9 73 ± 1%) and CTRL MN, suggestive of an additive neurotoxic impact of mC9-MG on mC9-MN (Fig. 8E and fig. S15H). In mC9-MG:isoC9-MN coculture, we also found reduced survival of isoC9-MN (42 ± 3.38%) (Fig. 8E) when compared to isoC9-MG:isoC9-MN (73 ± 1%); conversely, we noted an increased survival of mC9-MN (51 ± 1.1%) when cocultured with isoC9MG versus mC9-MG (23 ± 3.46%) (Fig. 8E). These results suggest that, following AMPA stimulation, mC9-MG drive enhanced MN death irrespective of genotype and that the increased death of mC9-MN in mC9MG-MN coculture is driven by mC9-MG. In contrast, MG numbers were comparable between the mutant versus iso cocultures and CTRL cocultures (fig. S15, D and I). Together, these findings suggest that the increased activation of mC9-MG following AMPA stimulation in MG-MN coculture leads to enhanced mC9MN death.

Next, we tested whether MG-mediated increased neuronal injury following AMPA treatment reflects sustained immune activation owing to disrupted autophagy by measuring NLRP3, NF-κB, and the autophagy marker p62 in cocultured microglial cells following AMPA stimulation. Increased NLRP3 and nuclear NF-κB expression alongside a concomitant reduction of the p62 level was seen in mC9-MG compared to isoC9-MG and CTRL-MG in the genotype-matched MG-MN cocultures following AMPA treatment (Fig. 8, F and I, and fig. S15, J to L). Conversely, in isoC9MG:mC9MN coculture, we noted a proportional increase of p62^+^ puncta following AMPA stimulation (Fig. 8G). We also noted reduced p62 puncta following AMPA stimulation in mC9-MG even when cocultured with isoC9-MG, showing that disrupted autophagy is a cell-autonomous deficit of mC9-MG (Fig. 8G). To test whether boosting autophagy would increase MN survival, we treated isolated MN and cocultures of MG-MN with rapamycin. Rapamycin treatment resulted in reduced IL-1β and increased mC9 MN survival in mC9-MG:mC9-MN and mC9-MG:isoC9-MN cocultures, but not in isolated MN cultures (Fig. 8, D and E, and fig. S15, H and C). We also noted an increase in the number of p62^+^ puncta following rapamycin treatment in mC9-MG:mC9-MN and mC9-MG:isoC9-MN cocultures, thereby indicating the activation of autophagy in mC9-MG when cocultured with MN (Fig. 8G and fig. S16). Concomitantly, we observed the reduced activation of NLRP3 inflammasome and NF-κB signaling in mC9-MG in MG-MN cocultures in the presence of rapamycin (Fig. 8, H and I). Together, these findings suggest that disrupted microglial autophagy in mC9-MG contributes to MN death following neuronal excitotoxicity and that boosting microglial autophagy reduces MN death.

### Blood-derived macrophages from *C9orf72* carriers display an activated phenotype and impairment of phagocytosis

**Fig. 9.**
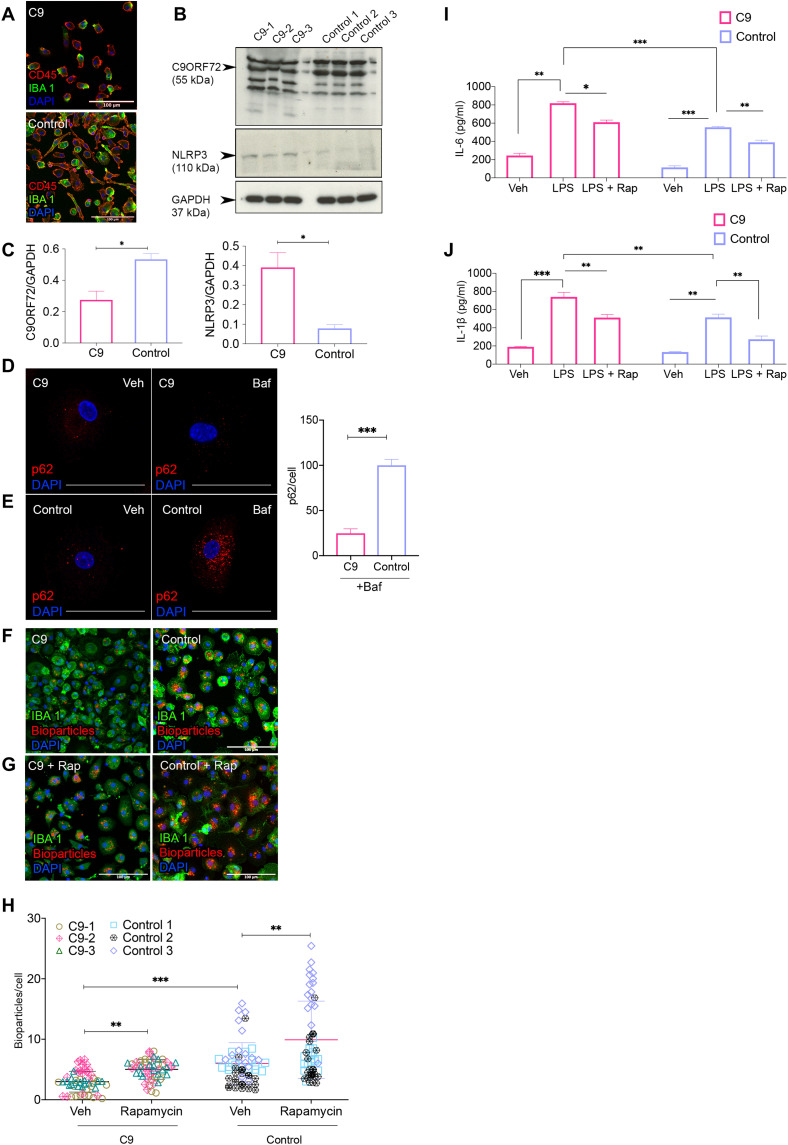
Blood-derived macrophages isolated from people with the *C9orf72* mutation recapitulate deficits observed in mC9-MGs. (**A**) Representative images of immunofluorescence staining for CD45 and IBA-1 in C9-carrier and control PBMC-derived macrophages. Scale bars, 100 μm. (**B**) Immunoblot and (**C**) densitometric quantification demonstrating the reduced abundance of C9ORF72 (~55 kDa) (graph, left) and increased levels of NLRP3 (~110 kDa) (graph, right), in three C9 macrophage samples compared to control macrophages. Data are represented as means ± SD, and statistical analysis was performed using Student’s *t* test. *P < 0.05. (**D** and **E**) Representative images of immunofluorescence staining of p62-positive puncta after 6 hours of DMSO (vehicle control) and bafilomycin treatment. Scale bars, 50 μm. Graph demonstrating significantly fewer p62-positive puncta in C9 macrophages compared to control; statistical analysis was performed by Student’s *t* test. (**F** and **G**) Representative images of immunofluorescence staining showing phagocytosis assay performed with pH-sensitive zymosan bioparticles in C9 macrophages and control macrophages at 120 min [IBA-1 (green) and zymosan bioparticles (red)]. Scale bars, 50 μm. (**H**) Quantification of the number of internalized zymosan bioparticles at 120 min in C9 macrophages and control macrophages showing reduced phagocytosis in C9 macrophages; statistical analysis was performed using two-way ANOVA and Tukey’s multiple comparisons test (**P < 0.01 and ***P < 0.001), and data represent means ± SD across the pooled data from three C9-ALS cases with age-/sex-matched control. (**I** and **J**) ELISA for proinflammatory cytokines (IL-6 and IL-1β) across C9 macrophages and control macrophages in unstimulated and LPS-stimulated condition in the presence and absence of rapamycin, demonstrating exaggerated immune response in C9 macrophages following LPS stimulation. Statistical analysis was performed using two-way ANOVA and Tukey’s multiple comparisons test (*P < 0.05, **P < 0.01, and ***P < 0.001). Data are represented as means ± SD.

To further assess the disease relevance of our iPSC findings, we next directly studied blood-derived macrophages from volunteers with the *C9orf72* mutation and their respective age- and sex-matched controls (Table 1). We isolated peripheral blood mononuclear cells (PBMCs) and differentiated them into macrophages by treatment with M-CSF (80 ng/ml) for 7 days (CD45^+^ and IBA1^+^) (Fig. 9A). Quantitative immunoblot revealed the reduced abundance of C9ORF72 in *C9* mutation–derived macrophages (Fig. 9, B and C). Concomitantly, we also observed significantly higher levels of NLRP3 in blood-derived macrophages derived from C9-positive subjects (Fig. 9, B and C) and the increased localization of p65 (one of the five components that form the NF-κB transcription family) in IBA1^+^ cells in postmortem spinal cord tissue from C9-ALS cases (fig. S17, B and C), thereby confirming the proinflammatory state of C9-ALS myeloid cells. Furthermore, we noted the reduced accumulation of p62-positive puncta following bafilomycin treatment in C9 macrophages when compared to their respective age- and sex-matched controls (Fig. 9, D and E, and fig. S17A), suggestive of disrupted autophagy in C9-positive blood-derived macrophages. C9 macrophages also secreted significantly higher levels of the proinflammatory cytokines IL-6 and IL-1β following LPS treatment (Fig. 9, I and J). We next examined phagocytosis. Macrophages from *C9*-positive subjects demonstrated a phagocytic deficit with significantly reduced zymosan bioparticle uptake over a period of 2 hours (Fig. 9, F to H). Notably, induction of autophagy in C9 macrophages by rapamycin treatment resulted in reduced IL-6 and IL-1β expression, as well as amelioration of the phagocytosis impairment (Fig. 9, F to J). Collectively, these findings are consistent with a *C9* mutation–dependent dysregulation of autophagy in macrophages and MG, resulting in an altered but reversible immune activation state.

**Table 1.** Clinical demographics of cases and controls for the PBMC-derived macrophage studies. F, female; M, male.

ID	Genotype	Diagnosis	Sex	Ethnicity	Age of onset (years)	Age at blood sample (years)	Disease duration (months)	Site of onset	Drug history	Family history
Control-1	Control	–	F	Caucasian	–	51	–	–	No	No
Control-2	Control	–	M	Caucasian	–	42	–	–	No	No
Control-3	Control	–	F	Caucasian	–	78	–		Thyroxine	No
C9-1	*C9orf72*	ALS	F	Caucasian	52	54	24	Lower limb	Riluzole	FTD
C9-2	*C9orf72*	Asymptomatic carrier	M	Caucasian	–	44	–	–	No	ALS/FTD
C9-3	*C9orf72*	FTD	F	Caucasian	76	77	84	–	Amlodipine, atorvastatin	FTD

## DISCUSSION

C9ORF72 is expressed in human myeloid cells, but its function in human immune cells has only been partially explored (*13*, *35*). Through the combination of patient iPSC-derived MG, isogenic controls, and patient-derived macrophages, we show that mutant myeloid cells are deficient in phagocytosis and are hyperresponsive to inflammatory stimuli. Furthermore, using a coculture of MNs and MG, we demonstrate that the *C9orf72* mutation confers a cell-autonomous deficit in autophagy leading to a hyper-activated NF-κB and NLRP3 axis, which contributes to enhanced MN death.

Multiple lines of evidence including p62^+^/ TDP-43 inclusions in patient autopsy samples and identification of ALS causal genes implicated in the autophagy pathway suggest an important role for autophagy dysregulation in ALS (*36*). Although severalstudies have examined the consequences of C9ORF72 loss-of-function on neuronal biology and function (*37*, *38*), haploinsufficiency of C9ORF72 has not previously been investigated at a cellular level in *C9orf72* patient iPSC-derived MG/myeloid cells. A range of studies suggest interaction of C9ORF72 at several points with the autophagy pathway. These include p62-dependent recruitment of cargo, followed by the trafficking of ULK-1 autophagy initiation complex for autophagosome formation through its interaction with Rab proteins and involvement of C9ORF72 in later stages of autophagy during the fusion of the autophagosome with the lysosome (*39*). At the cellular level, C9ORF72 forms a stable multifunctional complex with SMCR8 and WDR41 to execute these autophagic and vesicle trafficking events (*40*, *41*). This was further supported by independent and combinatorial knockdown of *C9ORF72* and *SMCR8*, revealing their role in the initiation of autophagy; however, they may have opposing roles in the regulation autophagy flux (*27*). Further studies are required to understand the precise role of C9ORF72 in regulating autophagy in immune cells.

So far, autophagy and the C9ORF72 interactome have largely been studied from a neurocentric perspective (*27*). However, the role of C9ORF72 in microglial autophagy and the impact of mutation-dependent dysregulation of autophagy on microglial biology are unclear. Our study emphasizes the value of studying cell type–specific behavior, not only to uncover previously unidentified interacting partners, but also to address nonneuronal consequences of dysregulated autophagy. We report a previously unknown interaction of C9ORF72 with UFM 1 (ubiquitin fold modifier 1) and UFL1 (an UFM1 E3 ligase), which is essential for hematopoietic stem cell function, as well as the transcription factor PU.1 that collectively suggest additional roles for C9ORF72 in myeloid cell function (*42*). UFMylation has been recently shown to be involved in ER-phagy, thereby providing indirect evidence for the role of C9ORF72 in UFMylation-induced ER-phagy (*43*). Furthermore, an elevated interferon response to proinflammatory stimuli, such as LPS, is observed in UFMylation-deficient cells, similar to the finding in C9 knockout (KO) mice and suggestive of a mutually dependent mechanistic outcome (*13*, *44*). The finding that C9ORF72 in hiPSC-derived MG-like cells is a binding partner of key mediators of the autophagy initiation complex, such as SMCR8 and WDR41, is consistent with previous reports (*26*, *27*). Rodent studies from *Smcr8^−/−^* and *Wdr^−/−^* mice also exhibited an exaggerated immune response similar to our findings of human mC9orf72 myeloid cells upon LPS stimulation. This deficit was reversed by the KO of endosomal TLRs consistent with SMCR8 negatively regulating TLR signaling. Together, this suggests that functional loss of the C9ORF72-SMCR8-WDR41 complex drives an altered immune state through reduced autophagy (*45*). However, TLRs are not the only immune responders reliant on endolysosomes for their activity; other inflammatory mediators, such as NLRP3 and members of NF-κB pathway, are also selectively degraded by autophagy (*46*). The NLRP3 inflammasome is a cytosolic multiprotein complex, which responds to cellular stress/endogenous stimuli, like secreted ATP and reactive oxygen species, and promotes the activation of IL-1β and IL-18 through active caspase-1 (*47*, *48*). Furthermore, NF-κB upon exposure to inflammatory stimuli is activated via signaling cascades including the IKK kinase complex, resulting in nuclear translocation of NF-κB to initiate the transcription of proinflammatory cytokines (*49*). Excitotoxicity, a key pathomechanism in ALS, has been reported to elicit microglial activation (*12*); however, it was not known whether the enhanced vulnerability of *C9orf72*-ALS MNs to excitotoxicity would result from microglial activation in *C9orf72*-ALS and whether such activation would play a causal role in MN death. Proinflammatory cytokines are known to elicit neuronal death via nitric oxide and free radicals (*50*). Given that autophagy regulates the NLRP3 inflammasome complex and IKKα/β kinases, which result in the activation of the NF-κb pathway (*51*), our pulse-chase experiments using LPS on microglial monocultures and AMPA (excitotoxic stimulus) on cocultures of MG-MN revealed persistent activation of NLRP3 inflammasome and NF-κB signaling in mC9-MG, leading to the increased production of cytokines in the absence of autophagy-mediated feedback inhibition. This result suggests that cell-intrinsic vulnerability of mC9-MG to triggering stimuli contributes to enhanced MN death in *C9orf72*-ALS. Our finding of increased NLRP3 activation and reduced autophagy activation in human mC9-MG is in line with the existing literature reporting elevated levels of NLRP3 in astrocytes in sporadic ALS cases and suboptimal autophagy in the ventral horn of patients with ALS (*48*, *52*, *53*).

Treatment with rapamycin can confer neuroprotection through pathways independent of autophagy (*54*) and has been shown to directly improve the survival of sporadic ALS-MN against an excitotoxic stimulus (*55*). However, our results suggest that the neuroprotective effect of rapamycin on mC9-MN is mediated through boosting microglial autophagy in MG-MN cocultures, as rapamycin had no direct effect on mC9-MN vulnerability to AMPA-mediated excitotoxicity (fig. S15C). Future experiments using selective genetic manipulation of autophagy in microglial cells from MG-MN cocultures will help to delineate autophagy-specific effect of rapamycin. Besides rapamycin, the increasing identification of a range of drugs that appear to boost autophagy may be of relevance, not only to *C9orf72*-ALS, but also to many other neurodegenerative diseases (*54*, *56*–*59*).

Emerging evidence suggests a cross-talk between autophagy and phagocytosis. This includes recent findings demonstrating that LC3-associated phagocytosis in myeloid cells is an efficient intracellular eliminator of engulfed extracellular cargo, including misfolded proteins (*60*, *61*). Our finding of reduced phagocytosis in m*C9orf72* myeloid cells that is ameliorated by boosting autophagy is consistent with these reports. The relevance of our findings for the accumulation of TDP-43 in *C9orf72*-ALS should be explored further, also with regard to potential TDP-43 spread. MG depletion has been reported to facilitate axonal spread of TDP-43 (*62*). Recent evidence also suggests an important role for LC3-associated endocytosis in MG for tau pathology and neuronal death (*63*).

While our findings are consistent with a loss-of-function model, our data do not exclude a combined loss- and gain-of-function mechanism for mutant C9ORF72 neurotoxicity. Such a two-hit model is supported by studies in transgenic mice and human neurons in vitro, showing that reduced levels of C9ORF72 lead to impaired autophagy and dipeptide repeat protein accumulation (*14*, *25*).

In conclusion, our study uncovers the impact of impaired autophagy on m*C9orf72* human microglial function, resulting in sustained activation of the NLRP3 inflammasome and NF-κB signaling pathways, which leads to an enhanced MN death following excitotoxic insult. This suggests a state of enhanced vulnerability of m*C9orf72* MG to triggering stimuli and cellular stress. Exogenous pharmacological activation/boosting of autophagy with mechanistic target of rapamycin inhibitors may ameliorate this proinflammatory phenotype, thus providing a target for translational research.

## MATERIALS AND METHODS

### Differentiation and characterization of MG (hiPSC-MG) from human iPSC

A control iPSC line (CTRL), three *C9orf72*-ALS/FTD patient iPSC lines (mC9-1, mC9-2, and mC9-3) harboring the GGGGCC repeat expansion, and their respective isogenic control lines (isoC9-1, isoC9-2, and isoC9-3) used in this study were generated with full Ethical/Institutional Review Board approval at the University of Edinburgh, as previously reported by our group (*11*, *33*). iPSCs were maintained on a monolayer of irradiated mouse embryonic fibroblast (Thermo Fisher Scientific) in Essential 8 Medium (E8, Gibco) in plates coated with Matrigel Growth Factor Reduced Basement Membrane Matrix and were passaged at 80% confluence with collagenase (1 mg/ml)/ dispase (0.5 mg/ml) (Thermo Fisher Scientific). All iPSCs were confirmed to be sterile and mycoplasma-free, and G-banding was performed at regular intervals to exclude the presence of any acquired chromosomal abnormalities in the iPSCs.

Human microglia-like cells (hiPSC-MG) were generated following a protocol previously described by our group (*23*). Briefly, myeloid progenitors expressing CX3CR1, CD11b, and CD45 were derived from mesodermally patterned embryoid bodies cultured in myeloid precursor media containing M-CSF (100 ng/ml; PeproTech) and IL-3 (25 ng/ml; Gibco) for 4 to 6 weeks. These myeloid precursors were then differentiated to microglia-like cells (hiPSC-MG) on poly-d-lysine–treated gelatinised plates (0.1%) in the presence of IL-34 (100 ng/ml; PeproTech), GM-CSF (10 ng/ml; PeproTech), and line-matched NPC-CM, which was supplemented in an increasing gradient (10 to 50%) every other day. The hiPSC-MGs obtained from control (CTRL), C9orf72 mutant (mC9), and isogenic control (isoC9) were immune-stained with microglial signature markers, such as TMEM119, P2Y12, and PU.1, and their abundance was quantified using the CellProfiler software. To further test the authenticity of the iPSC-derived microglia, we performed quantitative polymerase chain reaction (qPCR) for MG signature genes—*TMEM119*, *HEXB*, and *P2Y12*. The catalog codes for the antibodies alongside working concentration and reagents are mentioned in table S4.

### Immunocytochemistry

hiPSC-MGs grown on coverslips were fixed in 4% paraformaldehyde (PFA; Agar Scientific) in 1× phosphate-buffered saline (PBS) for 10 min at room temperature. After fixation, cells were washed three times with 1× PBS, permeabilized with 0.1% Triton X-100 (Thermo Fisher Scientific) in 1× PBS for 5 min, and blocked with 3% bovine serum albumin (Europa Bioproducts) in 1× PBS for 1 hour at room temperature. Incubation with primary antibodies for 2 hours was followed by addition of appropriate secondary antibodies. The cells were then washed three times with 1× PBS, counterstained with 4′,6-diamidino-2-phenylindole (DAPI; Thermo Fisher Scientific), and mounted on to glass slides in FluorSave (Millipore). The slides were observed using a Zeiss LSM confocal microscope. The catalog codes for the antibodies and reagents are mentioned in table S4.

### RNA extraction, reverse transcription, and PCR

Cells were harvested and RNA was extracted using the RNeasy Mini Kit (Qiagen). Contaminating DNA was removed using the RNase-Free DNase Set (Qiagen). The RNA was reverse-transcribed using the RevertAid RT Reverse Transcription Kit (Thermo Fisher Scientific) according to the manufacturer’s protocol. Quantitative real-time PCR was performed with DyNAmo ColorFlash SYBR Green Master Mix (Thermo Fisher Scientific) on a CFX96 Real-Time PCR System (Bio-Rad). Primer sequences are reported in earlier publication (*23*).

### Flow cytometry

The myeloid precursors were collected from the supernatant, centrifuged, washed with 1× PBS, and were treated with 1% Fc block (Miltenyi Biotec) in 1× PBS for 10 min at room temperature, before incubating them with primary antibodies for 1 hour on ice. Samples were then washed twice with 1× PBS and transferred onto round-bottom polystyrene tubes (BD Falcon) until assessment. The stained cell samples were analyzed using a FACS LSR Fortessa (four lasers) flow cytometer (BD Biosciences), and the data were processed using FlowJo software.

### High-throughput imaging and analysis platform for the quantification of microglial phagocytosis

Microglial differentiation was performed in a 96-well black/clear bottom plate with optical polymer base (Thermo Fisher Scientific) for 12 days. pHrodo-conjugated zymosan bioparticles (Thermo Fisher Scientific) were added to the hiPSC-MGs at a concentration of 0.5 mg/ml, and the plate was immediately loaded into ImageXpress Micro 4 High-Content Imaging System, a high-throughput live imaging system maintained at 37°C and 5% CO_2_ to monitor a real-time uptake of zymosan bioparticles by the hiPSC-MGs. Before loading, hiPSC-MGs were costained with Hoechst 33342 (Thermo Fisher Scientific). The cells were imaged using a 20× objective at an interval of 15 min for 2.15 hours (135 min), roughly accounting for 6000 cells per line per time point per differentiation. All lines reported in this study had a minimum of three biological replicates.

To determine the phagocytic index of individual genotypes (mutant and isogenic control), images were analyzed using Definiens Developer XD. Nuclei were first detected using a fluorescence threshold to separate background pixels from Hoechst-stained objects. Any holes in objects were filled, and the objects were smoothened by shrinking and growing each object by 2 pixels. Objects were then classified as nuclei based on their size (excluding small and large objects) and their elliptical fit (only objects with an elliptical fit greater than 0.8 were classified as nuclei). The nuclei were then expanded by 50 pixels, and within this area, we used a contrast threshold to identify the borders of pHrodo objects. pHrodo objects were smoothened by shrinking and growing each object by 2 pixels and then filtered using a fluorescence threshold to remove any objects that were not significantly brighter than the background (>2-fold change). From each image, we calculated the number of nuclei (Hoechst-stained objects), the total number of spots (pHrodo bioparticles), and the data were plotted as beads/cell after normalizing the total number of spots with total number of nuclei.

### LPS induction and assessment of NLRP3 and NF-κB activation

hiPSC-MGs were stimulated with LPS (100 ng/ml; Sigma-Aldrich) for 8 hours and followed by a washout. Cell lysates were collected at 4, 8, and 12 hours after washout, respectively, to examine the dynamics of NLRP3 and p62 by Western blotting. For testing the dynamics of NF-κB signaling by immunochemistry, cells after LPS washout were fixed at 8 and 12 hours, respectively, and stained with anti–NF-κB (Abcam) and anti-LC3B (Enzo Lifesciences) and observed in a Zeiss LSM confocal microscope. The details of the dilution for individual antibodies are mentioned in table S4.

For experiment with secondary stimulus, both LPS-primed and untreated mC9-MG and isoC9-MG were stimulated with 1 mM ATP for 30 min, and NLRP3 intensity was first measured at 30 min and also following a washout interval of 12 hours.

For assessing NF-κB activation, the Carl Zeiss image files were imported into Imaris version 8.0.2; the nuclear region was identified using DAPI staining and the intensity of the nuclear NF-κB was measured in voxels using Imaris software. Results were exported from Imaris in CSV formats, and graphs represent the colocalized voxels of DAPI and nuclear NF-κB.

### Cytokine ELISA

The concentration of IL-6 and IL-1β in cell supernatants was measured using DuoSet ELISA Development Systems (R&D Systems). hiPSC-MGs generated from control iPSC (CTRL), *C9orf72* mutant iPSC (mC9-1, mC9-2, and mC9-3), and their respective isogenic (isoC9-1, isoC9-2, isoC9-3) in a 12-well dish were treated with LPS or vehicle control (1× PBS) for 8 hours, and following a 12-hour washout, the cell-free supernatants were collected from the cultures after a brief centrifugation at 2000 rpm for 5 min. The samples were then assessed in triplicate for IL-6 and IL-1β, and the concentration (picograms per milliliter) obtained was further normalized to the 100 μg of protein content (picograms per milliliter) in each condition for monocultures of MG and 500 μg of protein for MG-MN cocultures. The catalog codes for the enzyme-linked immunosorbent assay (ELISA) kits are mentioned in table S4.

### Coculture of MG and spinal MN and quantification of neuronal and microglial number in the MG-MN coculture

Enriched spinal MN culture was generated from *C9orf72* patient and isogenic iPSC lines using an established protocol (*11*, *33*). Briefly, iPSCs were dissociated into single cells using Accutase and neuralized using dual SMAD inhibition by transforming growth factor–β inhibitor (20 μM; SB431542, Tocris), bone morphogenetic protein-4 inhibitor (0.1 μM; LDN-193189, Selleckchem), and glycogen synthase kinase inhibitor/wnt activator (3 μM; CHIR-99021, Tocris). Following neuralization, spheres were patterned using retinoic acid (RA), Sonic Hedgehog Signalling Agonist and gamma-secretase inhibitor. The spheres were maintained as a suspension culture for 2 weeks. After 2 weeks, the spheres were dissociated using MN dissociation media and titration buffer (refer to the Resource table). A total of 30,000 cells were plated on 96-well plates and on glass coverslips in 24-well plates. These cells were cultured in MN neurotrophic factor (MN-NF) containing l-glutamic acid and 5-fluoro-2'deoxyuridine (U/FDU) on day 1 and thereafter MN-NF plus U/FDU medium for 3 weeks. Microglial cells were differentiated from the myeloid precursors and were lifted using Accutase for coculturing with MNs. After 2 weeks, 5000 microglial cells (hiPSC-MG) were added to the MNs and were cocultured for 1 week in 1× neurobasal, 1× antibiotic-antimycotic, 1× GlutaMAX, 1× minimum essential medium nonessential amino acid solution, 100 μM β-mercaptoethanol, 1× B-27 supplement, 1× N-2 supplement, 1 μM RA, 2.5 μM ascorbic acid, brain-derived neurotrophic factor (10 ng/ml), recombinant human glial cell line–derived neurotrophic factor (10 ng/ml), recombinant human ciliary neurotrophic factor (10 ng/ml) and animal-free recombinant human insulin-like growth factor 1 (10 ng/ml), and IL-34 (20 ng/ml), before challenging them with 100 μM AMPA for 24 hours. Following AMPA addition, the cells were fixed with 4% PFA and stained with βIII-tubulin, Ionized calcium binding adapter molecule 1 (IBA-1), p62, NLRP3, and NF-κB for further experiments and quantification. DAPI, βIII-tubulin, and IBA-1 as above were imaged using an ImageXpress microconfocal with a 20× plan Apo objective and nine fields of view per well. All lines reported in this study had a minimum of three biological replicates. A custom analysis module was designed with MetaXpress software to count the number of βIII-tubulin + neurons and the number of IBA-1 + MG based on their size and intensity above background. The catalog codes for the reagents and antibodies are mentioned in table S4.

### Western blot analysis

Western blot analysis was carried out according to an established protocol (*64*). Briefly, the cells were lysed in lysis buffer [20 mM tris-Cl (pH 7.4), 1 mM EDTA, 150 mM NaCl, 1% Triton X-100, 1 mM EGTA, and 1 mM phenylmethylsulfonyl fluoride]. The protein concentration was calculated using the BCA Protein Assay Reagent (23225, Pierce Thermo Fisher Scientific). Protein samples were separated by SDS–polyacrylamide gel electrophoresis (Thermo Fisher Scientific) and transferred to 0.25-μm polyvinylidene difluoride membranes (Millipore, Bedford, MA, USA). The membranes were blocked with 5% skimmed milk and incubated at 4°C overnight with primary antibodies followed by addition of secondary antibody conjugated with horseradish peroxidase. The protein band signals were detected by ECL chemiluminescence substrate (Amersham) and quantified by the ImageJ software. The catalog codes for the reagents and antibodies are mentioned in the table S4.

### Autophagy flux assay using mRFP-GFP-LC3 dual-reporter probe in hiPSC-MG

Monocultures of hiPSC-MGs were lifted with 1× Accutase (Merck) and transduced with lentivirus expressing mCherry-EGFP-p62 at a MOI (multiplicity of infection) of 100 to obtain a transduction efficiency of 80%. The transduced live cells were visualized and fluorescence images were captured after 48 hours using a Zeiss 710 confocal microscope, followed by the manual counting of GFP and red fluorescent protein (RFP) puncta per cell using the ImageJ software. Colocalized puncta for GFP and RFP are indicated as autophagosomes (as represented in yellow bar in Fig. 4D), and red puncta are considered as autolysosomes (as represented in red bar in Fig. 4D).

### Generation of C9 KO iPSC line

C9ORF72 KO was generated using the following strategy, two small guide RNAs (gRNAs) were used to delete a 100–base pair (bp) fragment in exon 2 of C9ORF72 gene. Biallelic deletion and following nonhomologous end-joining event resulted in an in-frame STOP codon, thus leading to premature termination of C9ORF72 protein translation. Experimentally, two single gRNAs (sgRNAs) (gRNA-1: 5′-CAACAGCTGGAGATGGCGGT-3′, gRNA-2: 5′-ATTCTTGGTCCTAGAGTA-3′*)* were cloned individually in pSpCas9(BB)-2A-Puro (PX459) as per (*65*). Control iPSCs (CNTR-1, 8 × 10^5^) at 70 to 80% confluence were dissociated into single cells using 1× Accutase (Merck) and were nucleofected with 2 μg of Cas9-gRNA-1, 2 μg of Cas9-gRNA-2, and 1 μg of pEGFP-Puro (for positive selection of transfected cells), using the P3 Primary Cell 4D-Nucleofector X Kit (Lonza, program CA-137) on a Lonza 4D-Nucleofector X Unit (Lonza). Transfected iPSCs were plated on to 1:30 Matrigel-coated dish and cultured using E8 media (Gibco) and Y-27632 RHO-associated protein kinase (ROCK) inhibitor (10 μM) (Tocris). Selection with puromycin (1 μg/ml; Thermo Fisher Scientific) was commenced 24 hours after nucleofection and continued for 24 hours. Cells were dissociated and plated at a low density (1 × 10^4^ cells per 10-cm plate) for clonal selection upon confluence. Ninety-six individual clones were manually picked and screened for 100-bp deletion in exon 2 using the following primers C9-KO_Fw: 5′-ACAGGATTCCACATCTTTGACT-3′ and C9-KO_Rev: 5′-GCGATCCCCATTCCAGTTTC-3′. The positive C9-KO iPSC clone was confirmed by Sanger sequencing (fig. S5A) and immunoblot analysis (fig. S5A), which validated the loss of C9ORF72 protein.

### Generation of EGFP-C9ORF72 knock-in iPSC line

sgRNAs were designed using the CRISPR design toolkit (http://crispr.mit.edu) and cloned into pSpCas9(BB)-2A-Puro (PX459) V2.0 plasmid (Addgene: #62988) (*65*). Sequences of sgRNAs are as follows: sgRNA (1): 5′-GCATTTGGATAATGTGACAGT-3′; sgRNA (2): 5′-GTCACATTATCCAAATGCTC-3′; sgRNA (3): 5′-GACCGCCATCTCCAGCTGTTG-3′. Genomic targeting efficiency was determined in the CTRL-1 iPSC line via T7 endonuclease I assay [New England BioLabs (NEB)]. The PCR amplicon flanking the *C9orf72* target site (generated using T7_Fwd and T7_Rev primers as listed in table S4) was denatured, reannealed, and subsequently digested. All three sgRNAs were effective in generating a double-strand break, and sgRNA (1) was taken forward because it introduces a double-strand break closer to the end of the 5′ untranslated region of the *C9orf72* gene locus.

A targeting vector comprising a sequence coding for an EGFP protein, flanked by ~1-kb homology arms (HAs), was cloned into pMTL23 vector backbone using Gibson assembly (*66*). HAs and EGFP incorporating a Kozak consensus sequence were generated through PCR amplification, using primers listed in the table S4. The pAAV-FLEX-EGFPL10a plasmid (Addgene) (*67*) was used to generate the EGFP fragment. Sequences of these fragments are listed in table S3. They were assembled by the NEBuilder HiFi DNA Assembly Cloning Kit (NEB).

CTRL-2 iPSCs at 70 to 80% confluence were dissociated into a single-cell suspension using 1× Accutase (Merck). A total of 8 × 10^5^ cells were nucleofected with 2 μg of Cas9-gRNA1 plasmid, 2 μg of targeting vector, and 1 μg of puromycin resistance vector (for positive selection of transfected cells) using the Lonza 4D-Nucleofector X unit (program CA137) following the manufacturer’s instructions. Transfected cells were plated onto Matrigel-coated (BD Biosciences) dishes in the presence of E8 medium supplemented with ROCK inhibitor (10 μM; Y-27632, Tocris). Twenty-four hours after nucleofection, cells were subjected to 24-hour puromycin selection (1 μg/ml). Upon confluence, cells were dissociated again into single cells and plated down at low density (2000 cells per 10-cm dish) for clonal selection. After 8 to 10 days, single colonies were isolated and transferred to Matrigel-coated (BD Biosciences) 96-well plates. Three hundred clones were picked manually. Locus-specific PCR was performed using C9Ext_Fwd (located in intron 1 of C9orf72, external to the 5′ HA) and C9_Rev (located in the 3′ HA within intron 2-3). The sequence of the primers is listed in table S4.

### Coimmunoprecipitation of EGFP-C9ORF72 and mass spectrometry

EGFP-C9ORF72 hiPSC-MGs were grown on 10-cm dish and were lysed using lysis buffer comprising: 20 mM Hepes (pH 7.5), 150 mM NaCl, 5 mM MgCl_2_, 10% glycerol, 0.5% NP-40, 10 mM sodium glycerophosphate, 10 mM sodium pyrophosphate, 0.1 μM microcystin-LR, 1 mM sodium orthovanadate, 100 nM guanosine 5'-O-[gamma-thio]triphosphate (GTPgS), and complete EDTA-free protease inhibitor cocktail. Three hundred fifty micrograms of protein from ctrl-MG and EGFP-C9ORF72-MG (two biological replicates and four technical replicates) was subjected to immunoprecipitation using 10 μl of Chromotek GFP-Trap beads. Samples were incubated on an end-to-end rotator in a cold room (4°C) for 90 min followed by the washes with lysis buffer. On-bead tryptic digestion followed by 8-plex tandem mass tag (TMT) labeling was performed, as described previously (*68*). TMT labeling was verified by taking 5 μl from each sample and was analyzed by liquid chromatography–tandem mass spectrometry (LC-MS/MS) analysis. TMT labeling efficiency was found to be >98%; the remaining samples were quenched by adding 5 μl of 5% hydroxyl amine. Samples were pooled and vacuum-dried. To increase the depth, the pooled TMT-labeled sample was fractionated using mini high-pH reverse-phase liquid chromatography (RPLC) strategy on home-made C18 stage tips (*68*), and eluted peptides were vacuum-dried until LC-MS/MS analysis.

### LC-MS/MS analysis

The basic RPLC fractions were reconstituted in 15 μl of 0.1% formic acid and 3% acetonitrile (ACN) buffer and subjected to LC-MS/MS/MS analysis on an Orbitrap Exploris 480 hybrid mass spectrometer that is interfaced with the 3000 RSLC nano-liquid chromatography system. Sample was loaded on to a 2-cm trapping column (PepMap C18 100A, 300 μm; part number: 160454; Thermo Fisher Scientific) at 5 μl/min flow rate using loading pump and analyzed on a 50-cm analytical column (EASY-Spray column, 50 cm–by–75 μm inner diameter (ID), part number: ES803) at 300 nl/min flow rate that is interfaced to the mass spectrometer using Easy nLC source and electrosprayed directly into the mass spectrometer. LC gradient was applied from a 3 to 25% of solvent-B at 300 nl/min flow rate (solvent-B: 80% CAN) for 100 min and increased to 45% solvent-B for 10 min and 40 to 99% solvent-B for 5 min which is maintained at 90% solvent-B for 10 min and washed the column at 3% solvent-B for another 10 min comprising a total of 145 min run with a 120-min gradient in a data-dependent MS2 mode. The full-scan MS1 was acquired at a resolution of 120,000 at mass/charge ratio (*m/z*) 200 between *m/z* 350 and 1200 and measured using ultrahigh-field Orbitrap mass analyzer. Precursor fit threshold of 70% at 0.7-Da isolation width filter enabled accurate isolation of precursor isotope envelope for the MS2 fragmentation. The top 10 precursor ions were targeted which are isolated using Quadrupole mass filter at 0.7-Da isolation width for the MS2 and fragmented using 36% higher-energy collisional dissociation analyzed using ultrahigh-field Orbitrap mass analyzer at a resolution of 45,000 at *m/z* 200. Automatic gain control (AGC) target for MS1 and MS2 was set at 300 and 100%, respectively, with maximum ion injection times of 28 ms for MS1 and 110 ms for MS2.

### Mass spectrometry data analysis and bioinformatics analysis

The MaxQuant software suite (*69*) version 1.6.10.0 was used for database search with the following parameter. Reporter ion MS2 type: 8-plex TMT with PIF (precursor intensity factor) of 0.7 to have accurate reporter ion intensities. The TMT isotopic reporter ion correction factors were manually entered as provided by the manufacturer. The following group-specific parameters were used: A built-in Andromeda search engine was used by specifying trypsin/P as a specific enzyme by allowing two missed cleavages, minimum length of seven amino acids, oxidation of (M), acetyl (protein N-terminal), and deamidation N and Q were selected as variable modifications. Carbamidomethylation Cys was selected as fixed modification. First search tolerance of 20 parts per million (ppm) and main search tolerance of 4.5 ppm were selected. Global Parameters: UniProt Human protein database (release 2017-02; 42,101 sequences) was used for the database search and 1% peptide and protein level FDR were applied. For protein quantification, min ratio count was set at 2 for accurate reporter ion quantification. The MaxQuant output protein group text files were processed using Perseus software suite (*70*), version 1.6.10.45 was used. The data were filtered for any proteins that are flagged as common contaminants and reverse hits. In addition, common background binders from Crapome database (*71*) were filtered, and lastly, two minimum unique peptides were retained for the downstream analysis. The TMT reporter ion intensities were log_2_-transformed, and subsequently, the TMT reporter tags of control and EGFP-C9ORF72 conditions were categorized to perform statistical analysis. Two sample Welch’s *t* test was performed by applying 1 and 5% permutation-based FDR to identify the differentially enriched and significant protein groups between EGFP-C9orf72 and control groups.

### *C9orf72* case and age- and sex-matched control cohort for PBMC collection

All clinical meta-data were collected as part of Scottish Motor Neurone Disease Register (SMNDR) and Care Audit Research and Evaluation for Motor Neurone Disease (CARE-MND) platforms (ethics approval from Scotland A Research Ethics Committee, 10/MRE00/78 and 15/SS/0216). All cases had corresponding whole-genome sequencing and diagnostic repeat-prime PCR, demonstrating pathogenic repeat lengths in the *C9orf72* locus through a commercially available kit (The AmplideX PCR/CE C9orf72 Kit) (*72*). Blood samples from cases and age- and sex-matched healthy volunteers were taken under Lothian NRS Human Annotated Bioresource ethics (15/ES/0094), in line with the Human Tissue (Scotland) Act 2006, after obtaining signed informed consent, as part of the Scottish Regenerative Neurology Tissue Bank.

### PBMC collection and macrophage differentiation

Venepuncture was performed on volunteers, and ~16 ml of blood was collected in two BD Vacutainer CPT Mononuclear Cell Preparation Sodium Citrate Tubes at the Anne Rowling Regenerative Neurology Clinic, University of Edinburgh. Samples were gently inverted ~5 times and were immediately centrifuged for 20 min at 1600*g* at room temperature, permitting the formation of a physical barrier between the mononuclear cells in plasma and the erythrocytes and granulocytes. Mononuclear cells were collected from the buffy coat as described in (*73*) and were differentiated to functional macrophages using M-CSF (80 ng/ml) in RPMI media (Lonza) for 7 days. These macrophages were then assayed for phagocytosis using zymosan beads (Thermo Fisher Scientific), and cytokine production (IL-6 and IL-1β) was measured across unstimulated and LPS-stimulated condition using ELISA (R&D Systems).

### Human postmortem tissue immunohistochemistry

We identified, from the Edinburgh Brain Bank, a cohort of *C9orf72*-ALS patients for neuropathological assessment. All clinical data were collected as part of SMNDR and CARE-MND platforms (ethics approval from Scotland A Research Ethics Committee, 10/MRE00/78 and 15/SS/0216), and all patients consented to the use of their data during life. Control brains were selected from the sudden death brain bank and therefore have not died of a chronic illness or neurological condition. All control cases are rigorously assessed by experts, both clinically and neuropathologically, to rule out any evidence of disease, with pathology being assessed using well-defined and published grading systems. All postmortem tissue was collected via the Edinburgh Brain Bank (ethics approval from East of Scotland Research Ethics Service, 16/ES/0084) in line with the Human Tissue (Scotland) Act 2006. The use of human tissue for postmortem studies has been reviewed and approved by the Edinburgh Brain Bank ethics committee and the Academic and Clinical Central Office for Research and Development medical research ethics committee.

Formalin-fixed, paraffin-embedded blocks of spinal cord tissue were cooled and sectioned on a Leica microtome in 4-μm sections on to a superfrost microscope slide. The following antibodies were used: (i) anti-IBA1 antibody (Abcam) and (ii) anti-p65 antibody (Thermo Fisher Scientific) and were stained using the Bond RX multiplex staining protocol as per the manufacturer’s instruction. Imaging was performed by a pathologist blinded to all clinical and demographic data.

### Statistical analysis

Statistical analysis was performed using Prism version 8.4.0 (GraphPad Software), and differences were considered significant when *P* < 0.05 (*P < 0.05; **P < 0.01; ***P < 0.001). For comparison of normally distributed data of two groups, two-tailed unpaired Student’s *t* test was performed. Comparisons of data consisting of more than two groups where they vary in two factors were performed using two-way analysis of variance (ANOVA) and subsequent Tukey’s and Sidak’s multiple comparisons test. Detailed statistical information for each figure, including statistical tests performed, is elaborated in the respective figure legends.

## Notes

View/request a protocol for this paper from *Bio-protocol*.
