# Supplementary material for: Cell-autonomous immune dysfunction driven by disrupted autophagy in C9orf72-ALS iPSC-derived microglia contributes to neurodegeneration
Source: Sci Adv. 2023 Apr 21;9(16):eabq0651. doi: 10.1126/sciadv.abq0651 (PMC10121169; doi:10.1126/sciadv.abq0651)
Supplement: Supplementary file 1 — Figs. S1 to S17 Tables S1, S3 and S4 Legend for table S2 [file sciadv.abq0651_sm.pdf]

Science Advances
AAAS

Supplementary Materials for

Cell-autonomous immune dysfunction driven by disrupted autophagy in *C9orf72*-ALS iPSC-derived microglia contributes to neurodegeneration

Poulomi Banerjee *et al.*

Corresponding author: Siddharthan Chandran, siddharthan.chandran@ed.ac.uk; Josef Priller, josef.priller@ed.ac.uk

Sci. Adv. **9**, eabq0651 (2023)
DOI: 10.1126/sciadv.abq0651

The PDF file includes:

Figs. S1 to S17
Tables S1, S3 and S4
Legend for table S2

Other Supplementary Material for this manuscript includes the following:

Table S2

Supplementary Fig 1 (Supplementary to Fig 1)

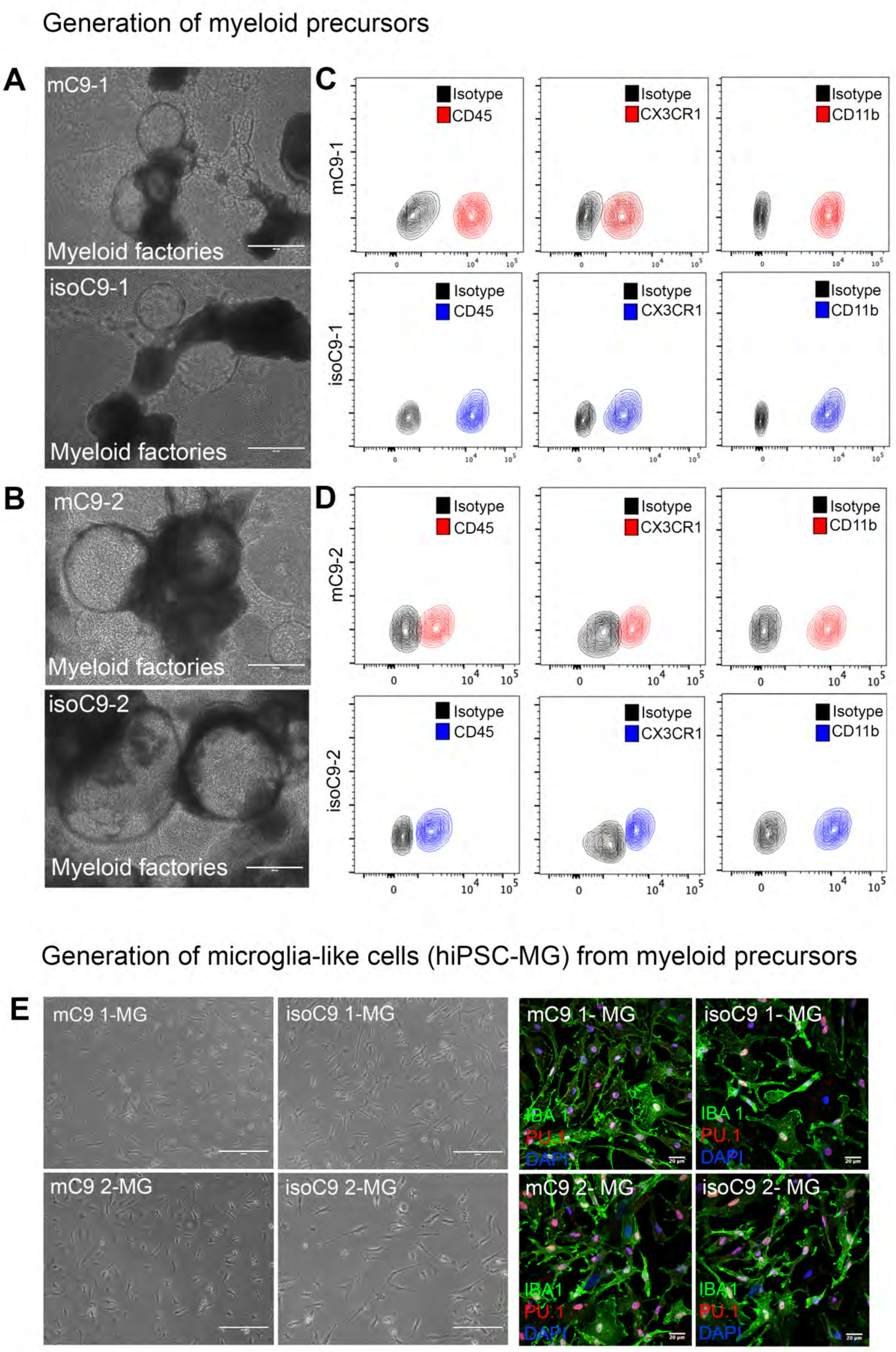

Supplementary Fig 1 - Generation and characterisation of human microglia-like cells (hiPSC-MG) from mC9 and isoC9 iPSCs: **(A, B)** Phase contrast images showing the formation of myeloid

factories from two pairs of C9 mutant (mC9-1 and mC9-2) and their respective isogenic iPSC lines (isoC9-1 and isoC9-2), Scale bar=200µm. **(C,D)** Myeloid precursors produced by the mC9 and isoC9 myeloid factories show comparable profile for key myeloid precursor markers- CD45,CX3CR1 and CD11b. (**E-left panel)** Phase contrast images of human microglia-like cells (hiPSC-MG) generated from myeloid precursors of mC9-1/isoC9-1 and mC9-2/isoC9-2 lines. **(E-right panel)** Representative immunofluorescence images of two pairs mC9-MG and isoC9-MG showing staining for IBA-1 and myeloid marker PU.1; scale bar=20 µm. Data representative of three independent biological differentiations from each iPSC line.

Supplementary Fig 2 (Supplementary to Fig 1)

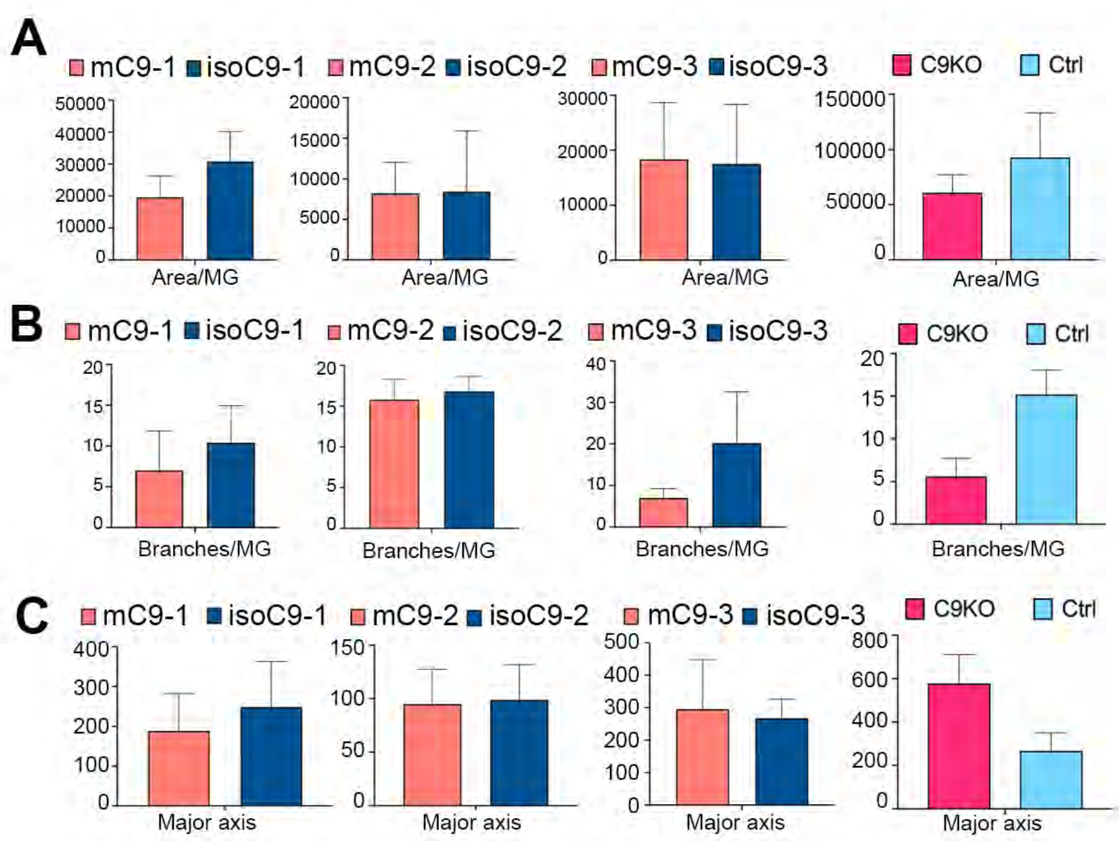

Supplementary Fig 2 - Morphometric analysis of mC9-MG, isoC9-MG, C9KO-MG and CTRL-MG: Morphometric analysis was performed using Cell Profiler software. Different parameters such as area per microglial cell (panel A), number of branches per microglial cell (panel B) and major axis of each microglial cell showed no significant differences across mC9-MG and isoC9-MG across three pairs and C9KO/CTRL-MGs. 30 cells per line from three independent biological differentiations were analysed.

Supplementary Fig 3 (Supplementary to Fig 2)

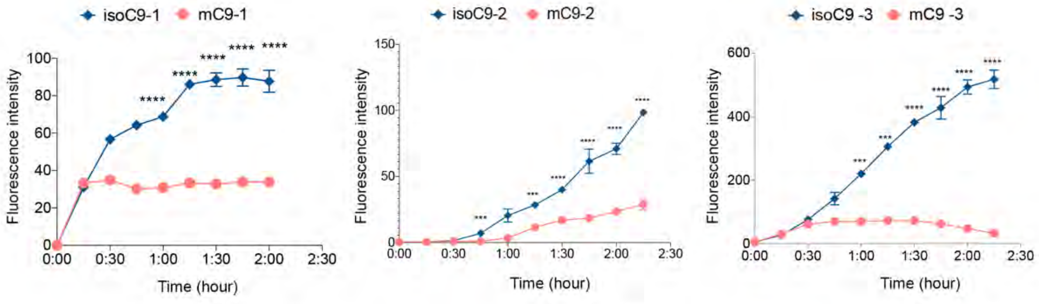

Supplementary Fig 3 - Reduced phagocytosis in mC9-MG: Graph showing real-time imaging of zymosan bioparticle uptake at 15 min intervals, demonstrating a phagocytic deficit in mC9-MG across three pairs of C9 mutant and C9 isogenic lines. Y axis in the graphs indicates the total fluorescence intensity of the internalised bioparticles per microglial cells. Statistical analysis was performed across mC9-MG and isoC9-MG using two-way ANOVA and Tukey's multiple comparison test; data are represented as mean +/- SEM; N=3 (*** $p \leq 0.001$; **** $p \leq 0.0001$). Data are representative of three independent biological differentiations from each iPSC line.

Supplementary Fig 4 (Supplementary to Fig 2)

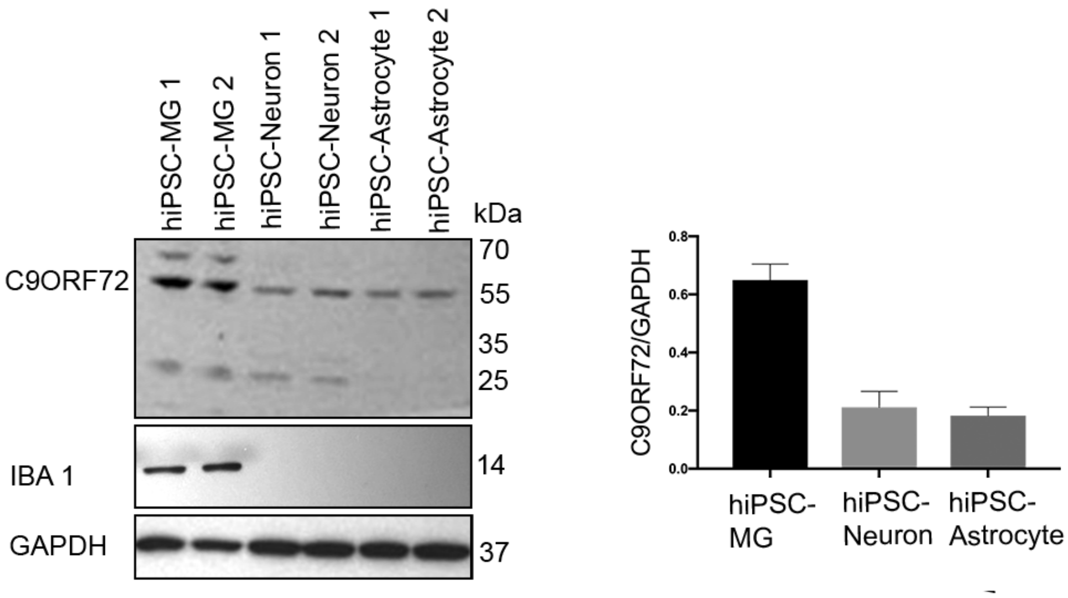

Supplementary Fig 4 - Comparison of C9ORF72 level across iPSC derived microglia (hiPSC-MG), neurons *(*hiPSC-neurons) and astrocytes (hiPSC-astrocytes): **(A)** Immunoblots showing the higher abundance of C9ORF72 in hiPSC derived microglia (hiPSC-MG) when compared to hiPSC derived neurons (hiPSC-neurons) and astrocytes (hiPSC-astrocytes) generated from two independent iPSC lines, immunoblot for IBA-1 confirms the identity of hiPSC-MG. **(B)** Bar graphs show the densitometric quantification of C9ORF72 levels over GAPDH for different cell types derived from iPSC, data represent mean +/-SD across 3 biological replicates.

Supplementary Fig 5 (Supplementary to Fig 3)

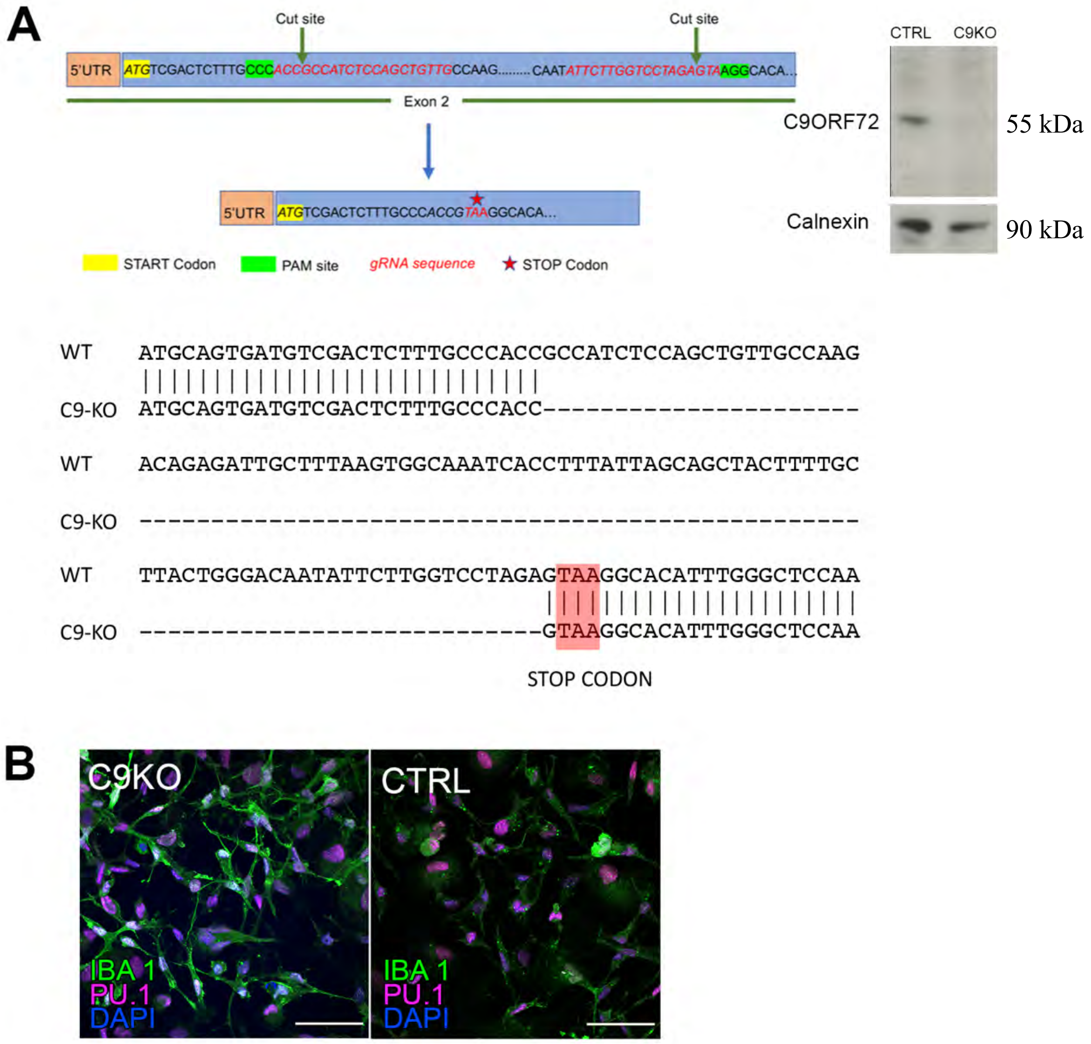

Supplementary Fig 5 - Generation and characterisation of C9KO-MG from C9KO iPSC line
(A) Strategy for the generation of C9 knock-out (C9KO) iPSC-line, two sgRNAs were used to delete a 100 bp fragment in exon 2, and following non-homologous end joining event, this resulted in the insertion of an in-frame STOP codon, immunoblot (right) confirming the absence of C9ORF72 protein in the C9KO iPSC line, Sanger sequencing depicting the deletion of 100bp region in exon2. Highlighted region shows the in frame stop codon. **(B)** Represents the immunostaining of C9KO iPSC derived microglia (C9KO-MG) and CTRL-MG confirming the positive staining for myeloid marker PU.1. Scale bar=20µm

Supplementary Fig 6 (Supplementary to Fig 3)

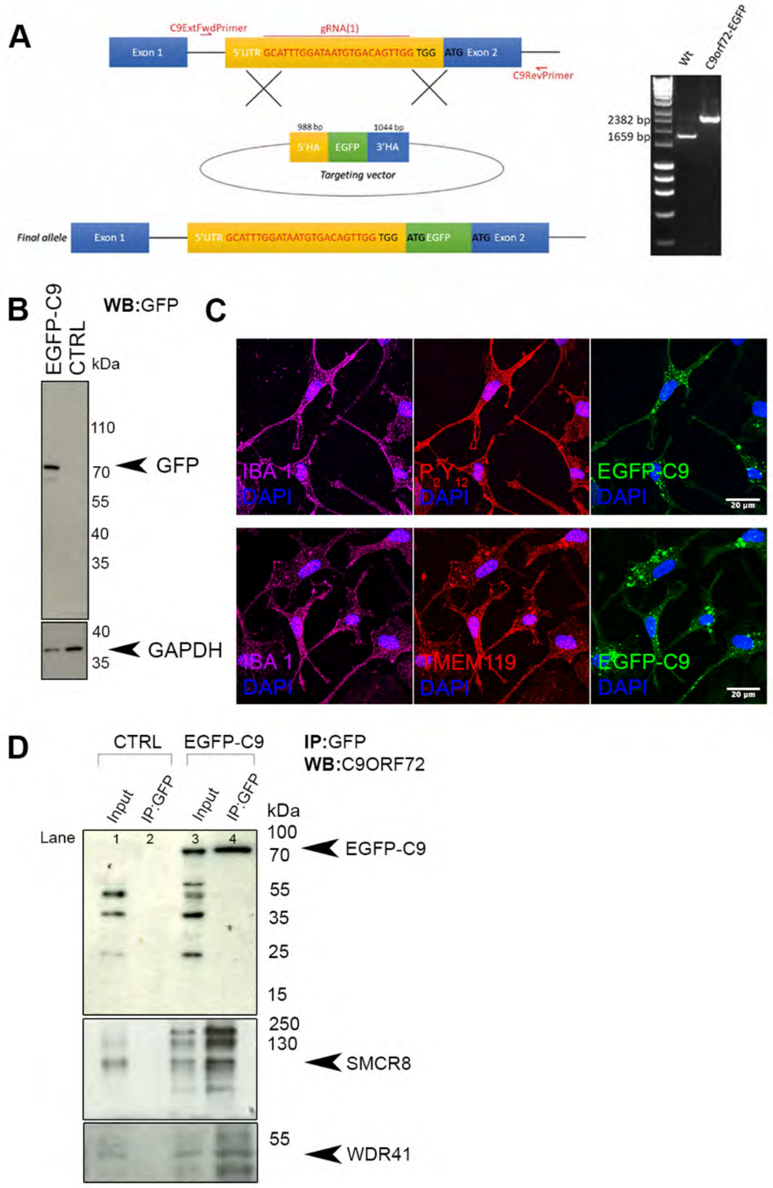

Supplementary Fig 6 - Generation and characterisation of EGFP-C9-MG from *EGFP-C9orf72* iPSC line and validation of the C9ORF72 interactome **(A)** Schematic diagram (left) depicting gene targeting strategy, and location of guide RNA (gRNA) and forward and reverse locus-specific primers used as part of the validation process for the homozygous clone (**see Supplementary Table 3** for sequences of fragments constituting the EGFP targeting vector and resource table for primer pair sequence). Homology arms (HAs) as shown: 5'HA comprises part of intron 1 and the 5'UTR of exon 1; 3'HA comprises exon 2 and part of intron 2. Agarose gel electrophoresis (right) showing genotyping screen using locus-specific primers. Agarose gel electrophoresis shows a single higher band for the homozygous EGFP-C9orf72 clone (2382 bp) when compared with the wild-type band (1659 bp). **(B)** Immunoblot demonstrating the expression of EGFP-C9 fusion protein in EGFP-C9-MG (corresponding to 70 kDa) as opposed to no bands in CTRL-MG. **(C)** Representative images of immunostaining for P2Y12 and TMEM119 confirming the identity of EGFP-C9-MG. Scale bar=20µm **(D)** Western blotting performed with C9ORF72 antibody for EGFP-C9ORF72 fusion protein (corresponding ~70 kDa in EGFP-C9-MG and 55 kDa in CTRL-MG), SMCR8 (105 kDa) and

WDR41 (48.5 kDa) in GFP pull down samples of EGFP-C9-MG and CTRL-MG confirming the interaction of C9ORF72 with SMCR8 and WDR41.

Supplementary Fig 7 (Supplementary to Fig 4)

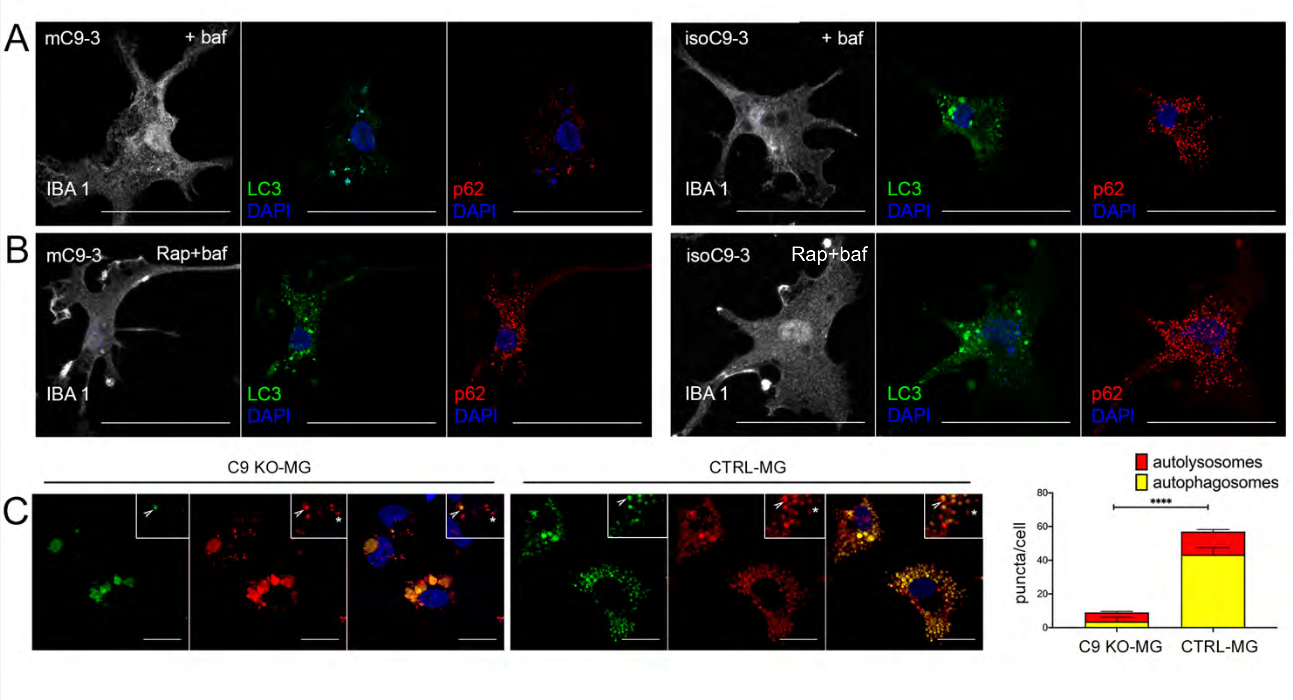

Supplementary Fig 7 - mC9-MG demonstrate a deficit in the initiation of autophagy (A) Representative immunofluorescence images demonstrating fewer p62 and LC3 puncta in mC9-3 as opposed to isoC9-3, scale bar = 50 µm. **(B)** Representative immunofluorescence images showing increase in the number of p62/LC3(+) ve puncta in presence of 10 µM rapamycin and 100 nM bafilomycin in m*C9* 3-MG and iso*C9* 3-MG. **(C)** Representative live imaging snapshots of mcherry-EGFP-p62 dual reporter probe transduced C9KO-MG and CTRL-MG; the white arrowhead in the inset represents autophagosomes (GFP+ve, mCherry+ve) and the white asterisk represents autolysosomes (GFP-ve, mCherry+ve). Stacked bar graphs (right) demonstrating the quantification of the autophagosomes and autolysosomes. Statistical analysis was performed using two-way ANOVA and Tukey's multiple comparison test for the number of autophagosomes across C9KO-MG and CTRL-MG. Data are represented as mean+/-SD across three independent differentiations from iPSC lines {n(C9KO- 15cells, CTRL-23cells)}, (*** $p \leq 0.001$) .

Supplementary Figure 8 (Supplementary to Fig 3)

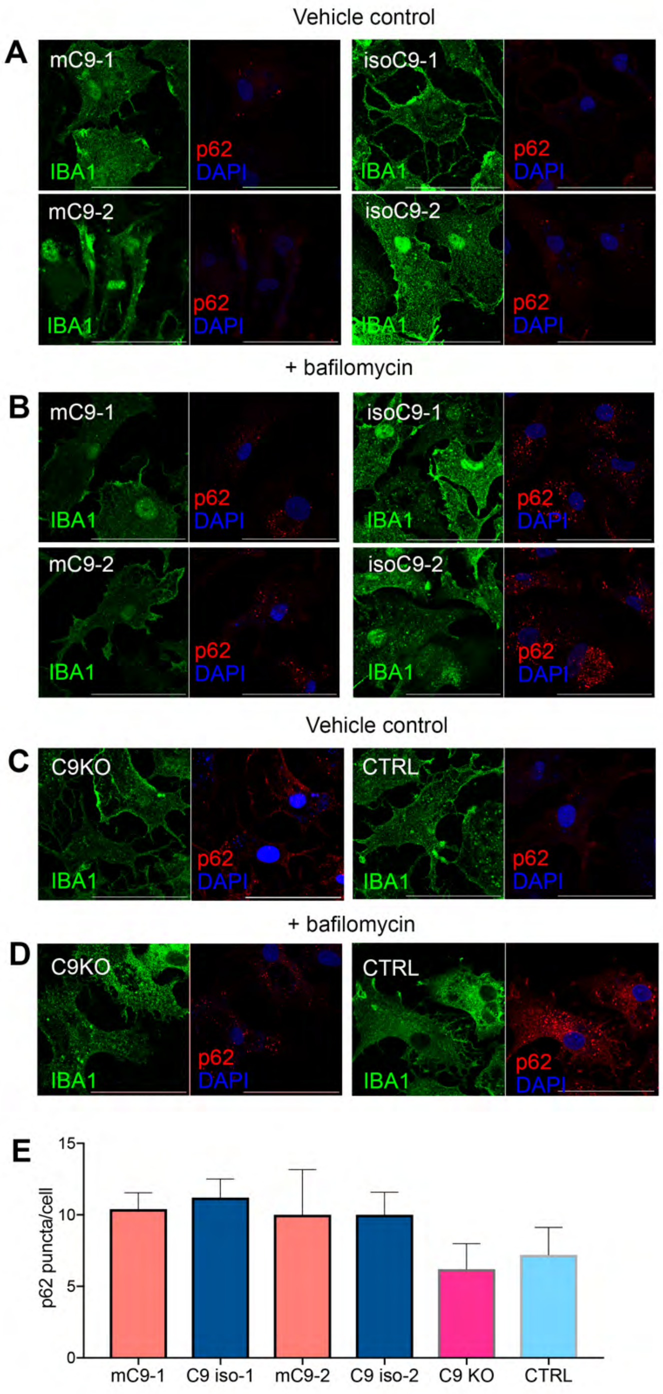

Supplementary Fig 8 – (A-D) Single channel images for IBA1 and p62 that have been considered for Fig 4A and Fig 4B. **(E)** Graph represents no change in the number of p62-positive puncta across mC9-MG and isoC9-MG and C9 KO-MG and CTRL-MG in vehicle-treated condition.

Supplementary Fig 9 (Supplementary to Fig 3)

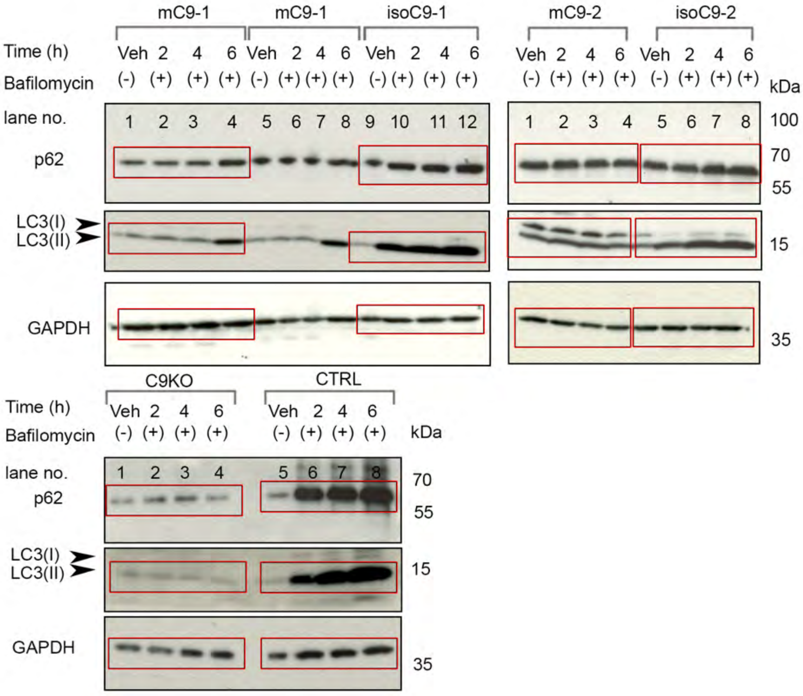

Supplementary Fig 9 - Full blot of Fig 3B
The bands highlighted in red rectangle have been considered for the main figure (Fig 3B).

Supplementary Fig 10

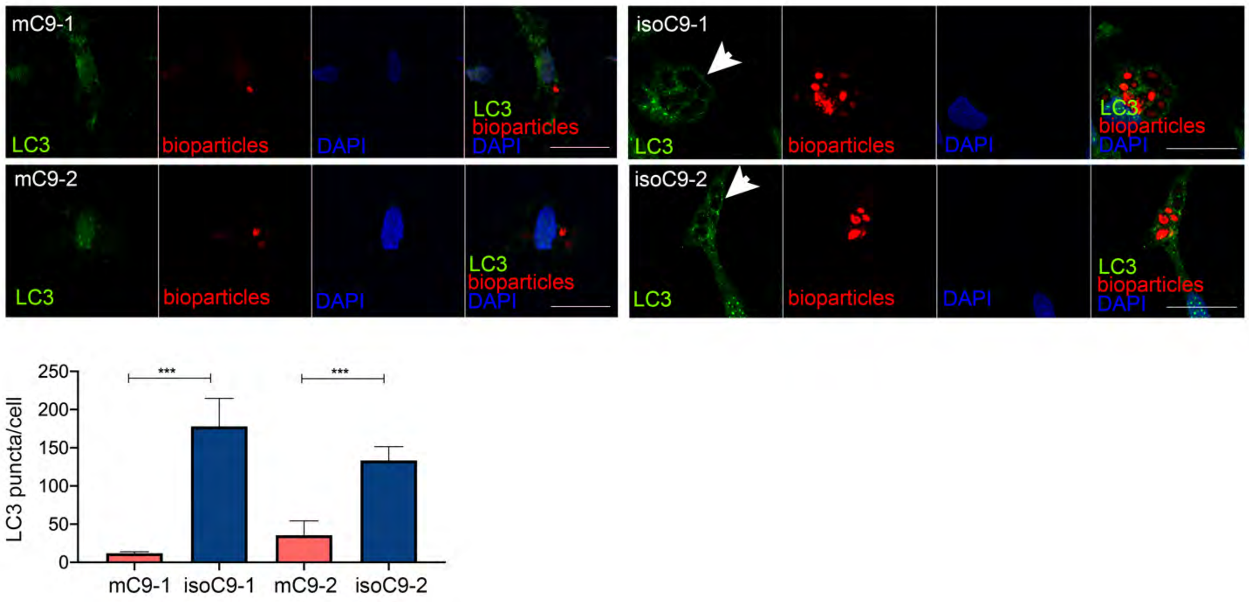

Supplementary Fig 10 - Appearance of LC3 puncta during zymosan bioparticle uptake assay: Zymosan uptake elicits the appearance of LC3 puncta (green) around zymosan bioparticles (red) in the isoC9-MG as opposed to mC9-MG, the number of LC3 puncta are quantified in graph (right), data

represents mean+/-SD, statistical analysis was performed by two way ANOVA and Tukey's multiple comparison test, N=3 (n=5) (*** $p \leq 0.001$). Scale bar=20µm

Supplementary Fig 11 (Supplementary to Fig 5)

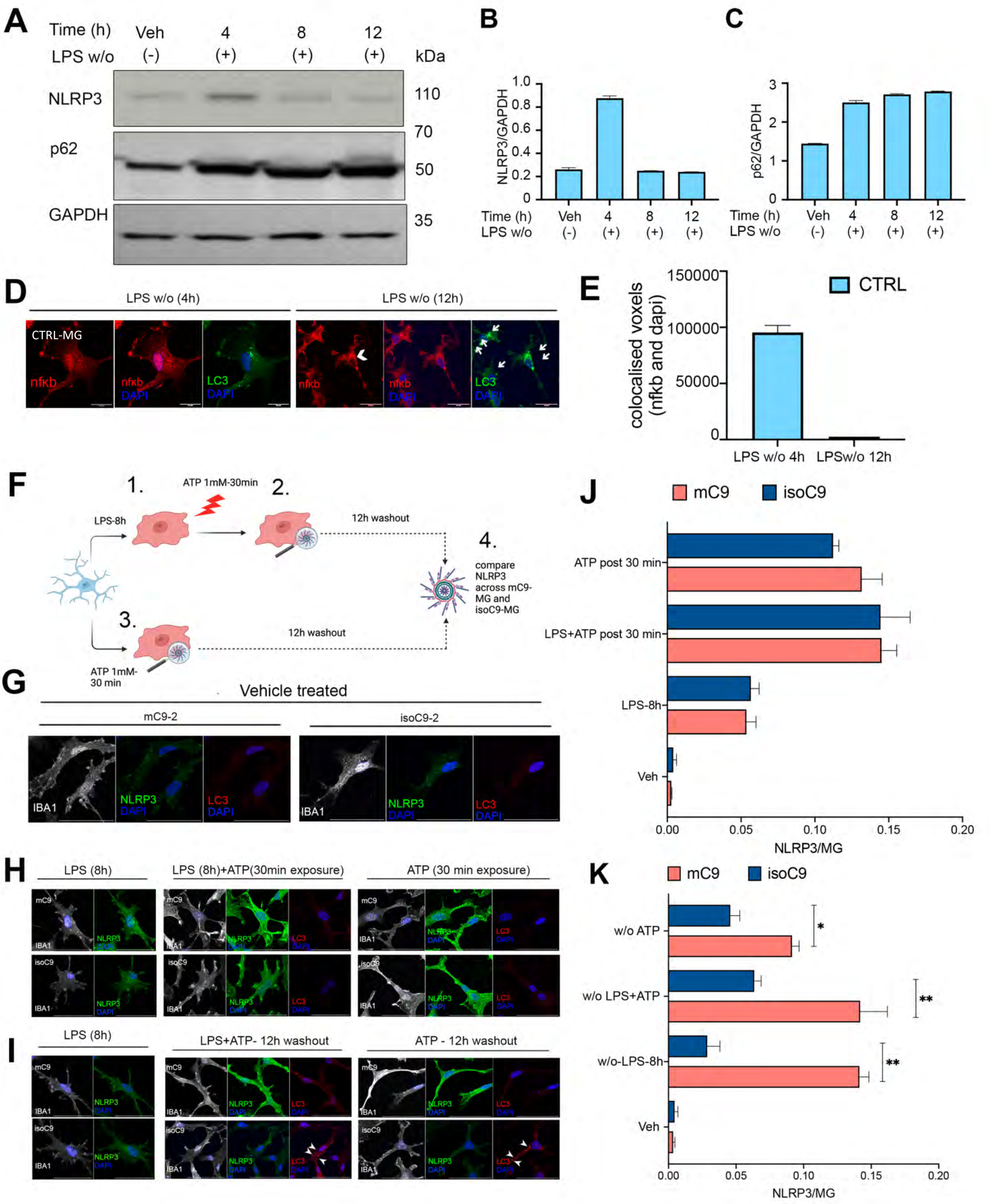

Supplementary Fig 11 - Assessment of NF-κb signalling and NLRP3 activation following LPS stimulation (A) Western blots showing immunoreactivity for NLRP3, p62 at 4 h, 8 h and 12 h post LPS washout and vehicle treated-1x PBS (veh) condition in CTRL-MG **(B, C)** Bar graphs showing densitometric values for NLRP3 and p62 normalised to GAPDH depicting a time-dependent decline of NLRP3 with a concomitant, reciprocal increase of p62 indicative of autophagy induction. **(D)** Representative images of immunofluorescence staining of NF-κB and LC3 demonstrating NF-κB signalling and autophagy activation at 4 h and 12 h post LPS washout in CTRL-MG. At 4h post LPS washout (left) CTRL-MG show nuclear localisation of NF-κB indicative of activated NF-κB signalling as opposed to the disappearance of NF-κB from the nucleus at 12 h post LPS washout indicative of an attenuation of NF-κB signalling, white arrow heads indicate the clearance of NF-κB from nucleus and white arrows indicate the appearance of LC3 puncta suggestive of autophagic activation at 12 h post LPS washout **(E)** Graph demonstrating the quantification of nuclear localisation of NF-κB as denoted by the colocalised voxels of NF-κB and DAPI; data is representative for 21 cells from three independent biological differentiation ; data represents mean +/-SD **(F)** Schematic representing experimental design to assess NLRP3 activation in mC9-MG and isoC9-MG by immunostaining following LPS, LPS+ATP and ATP exposure. Levels of NLRP3 and LC3 were measured in mC9-MG and isoC9-MG across all these treatment conditions at 30 minute time-point and following a 12 h washout period (w/o) **(G)** Immunofluorescence staining images representing the basal level of NLRP3 and LC3 in vehicle (DMSO) treated condition in mC9-MG and isoC9-MG. (**H**) Immunofluorescence staining images representing elevated level of NLRP3 in mC9-MG and isoC9-MG following an exposure to LPS , LPS+ATP and ATP at 30 minute time point (**I**) Immunofluorescence staining images representing elevated level of NLRP3 in mC9-MG at a 12 h washout time-point across LPS, LPS+ATP and ATP treated condition. Appearance of LC3 puncta (denoted by white arrow-heads) in isoC9-MG across both ATP and LPS+ATP treated condition is indicative of autophagy activation. **(J)** Graphs showing intensity of NLRP3/MG in vehicle treated, LPS, LPS+ATP and ATP treated condition at 30 min washout time-point across mC9-MG and isoC9-MG. (**K**) Graphs showing elevated intensity of NLRP3 in mC9-MG in LPS+ATP treated condition at 12 h washout time-point. 30 cells per condition across three independent biological differentiations per genotype was assessed through cell profiler software. Error bars represent +/- SD and (* $p \leq 0.05$ and ** $p \leq 0.01$)

Supplementary Fig 12 (Supplementary to Fig 5)

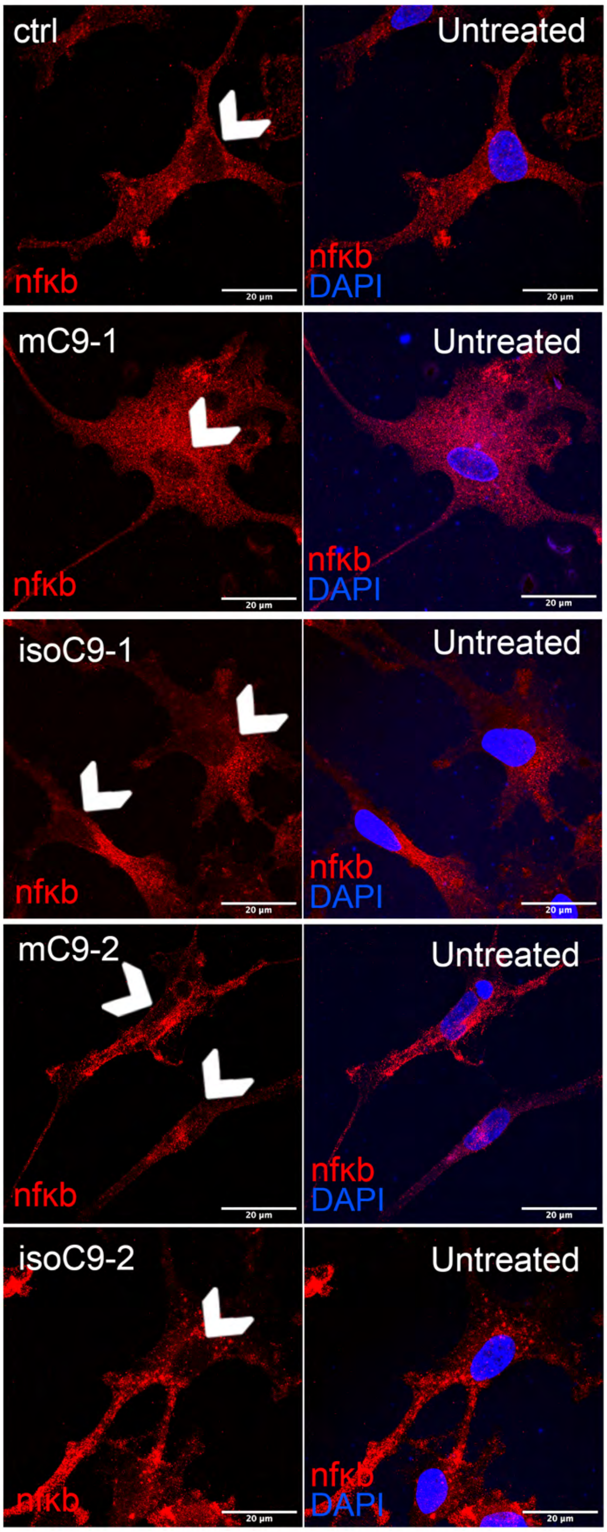

Supplementary Fig 12: Localisation of NF-κB in CTRL-MG, mC9-MG and isoC9-MG in unstimulated condition: Representative images of immunofluorescence staining showing the localisation of NF-κB at basal state across CTRL-MG, mC9 1-MG, isoC9 1-MG, mC9 2-MG and isoC9 2-MG. White arrow heads show the absence of nuclear NF-κB. Scale bar=20um

Supplementary Fig 13 (Supplementary to Fig 6)

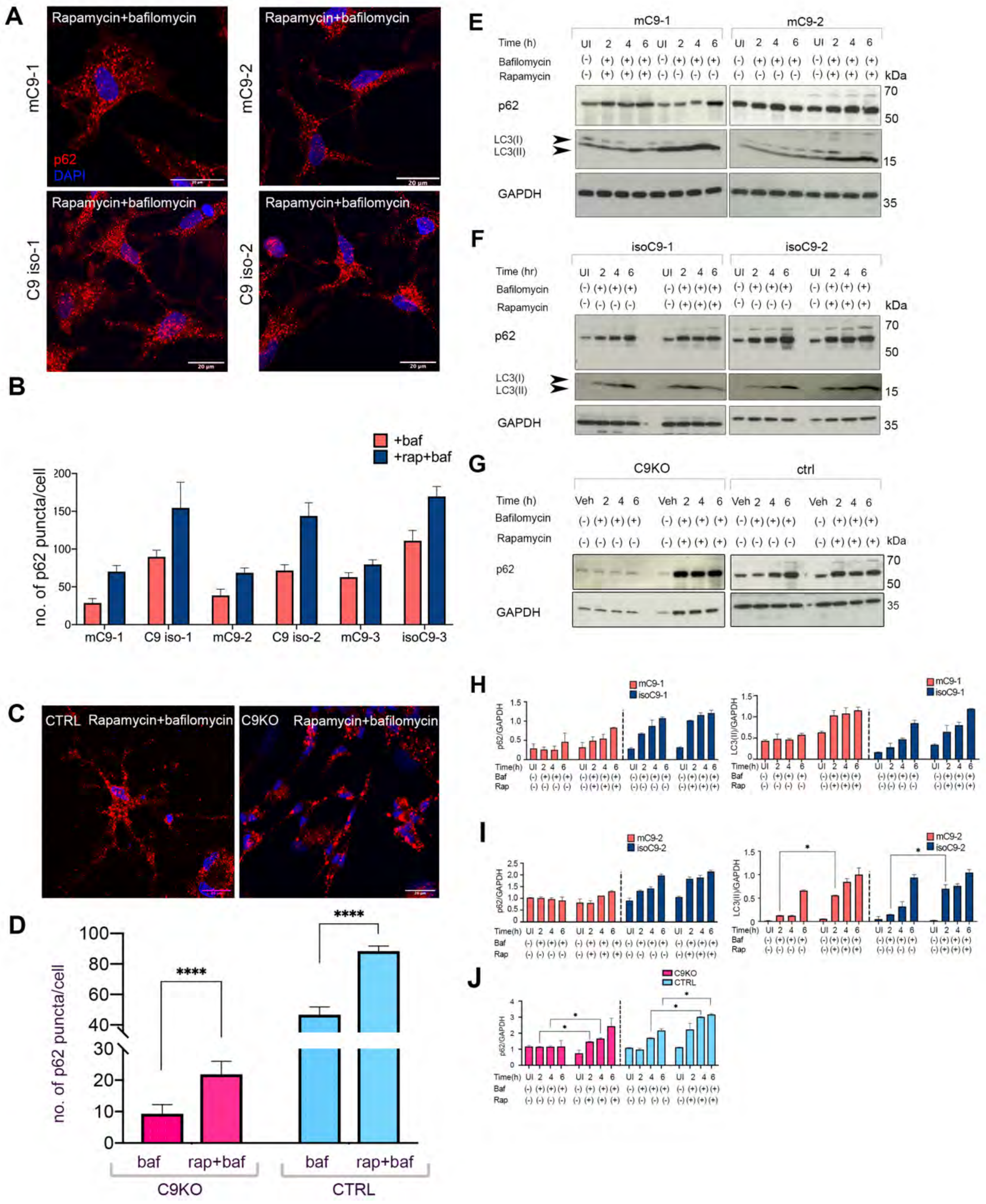

Supplementary Fig 13: Rapamycin induces autophagy in mC9-MG, isoC9-MG, C9KO-MG and CTRL-MG

(A) Representative images of immunofluorescence staining showing the increase in the number of p62(+) ve puncta in presence of 10 µM rapamycin and 100nM bafilomycin in m*C9* 1-MG, m*C9* 2-MG and iso*C9* 1-MG and iso*C9*2-MG Scale bar=20µm **(B)** Bar graph shows the quantification of p62 puncta from the immuno-fluorescence images across m*C9*-MG and iso*C9*-MG (3 pairs) depicting an increase in the accumulation of puncta in the mC9 -MG upon treatment with rapamycin, Statistical analysis was performed using two-way ANOVA and Tukey's multiple comparison test, (* $p \leq 0.05$; ** $p \leq 0.01$ *** $p \leq 0.001$). **(C)** Representative images of immunofluorescence staining of C9KO-MG and CTRL-MG showing the increased number of p62+ve puncta following rapamycin treatment at 6

h in C9KO-MG and CTRL-MG, **(D)** Bar graph depicting the quantification of the number of p62 puncta in C9KO-MG and CTRL-MG in presence rapamycin and bafilomycin, data is represented as mean +/-SD N=3 (n=10 cells were assessed across both genotypes). Statistical analysis was performed using two-way ANOVA and Tukey's multiple comparison test, **** $p \leq 0.0001$ **(E,F)** immunoblots showing the increased turnover of p62 and LC3(II)in both pairs of mC9-MG and isoC9-MG in presence of rapamycin **(G)** Immunoblots showing time dependent increase in the level of p62 in C9KO-MG and CTRL-MG following rapamycin treatment indicative of enhanced autophagy induction **(H,I)** Densitometric quantification of the level of p62, LC3 (II) over GAPDH shows the increment in the levels of p62 and LC3 post rapamycin treatment at 2 h,4 h,6 h in mC9 -MG, isoC9-MG. **(J)** Densitometric quantification of p62 levels depicting a time dependent increase of p62 level across C9KO-MG and CTRL-MG at 2 h,4 h,6 h post rapamycin treatment.

Supplementary Fig 14 (Supplementary to Fig 6)

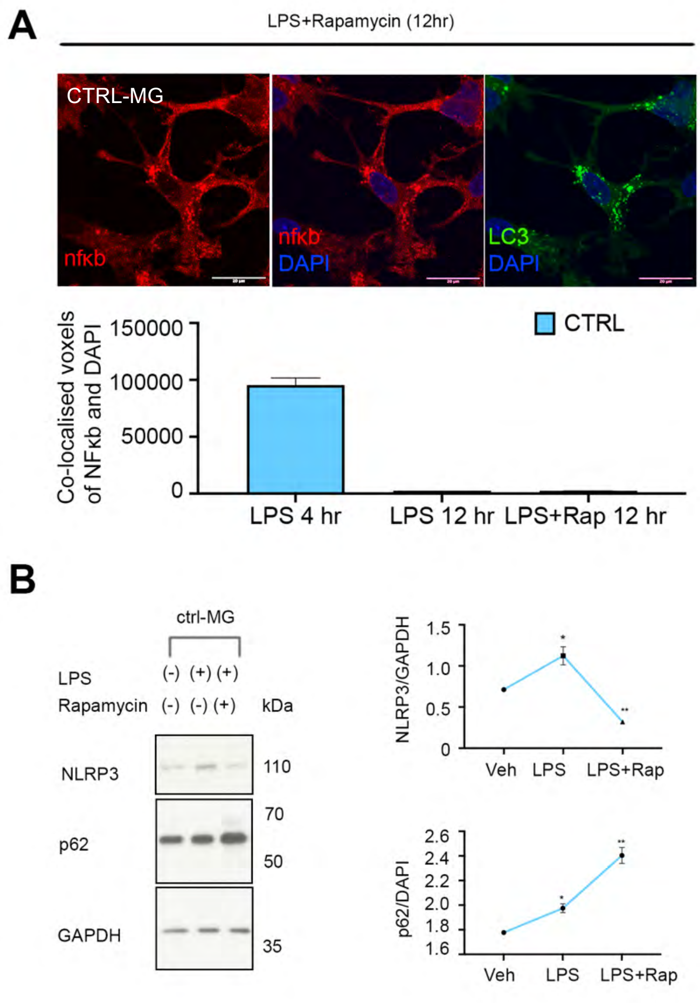

Supplementary Fig 14: Rapamycin dampens the effect of LPS and enables the attenuation of NF-κb signalling in CTRL-MG (A) Representative images of immunofluorescence staining showing the reduction of nuclear localisation of NF-κB in rapamycin-treated CTRL-MG, 12 hours post LPS washout. Scale bar=20µm. Quantification of the colocalization of NF-κb in the nucleus is shown in the bar graphs indicating a suppression of NF-κb signalling as a result of rapamycin treatment. Data is represented as mean +/-SD N=3 (n=20 cells were assessed) **(B)** Immunoblots and its quantification

(right) showing an increase in p62 immunoreactivity and a reciprocal decline in NLRP3 immunoreactivity in presence of rapamycin at 12 h. p-value was calculated using one-way ANOVA and multiple comparison analysis was performed using Tukey's multiple comparison test, data represents mean+/-SD, N=3 (** $p \leq 0.01$) N number for all experiments represents the number of times experiments were performed using cells generated from independent differentiations from iPSCs.

Supplementary Fig 15 (Suppl. To Fig 8)

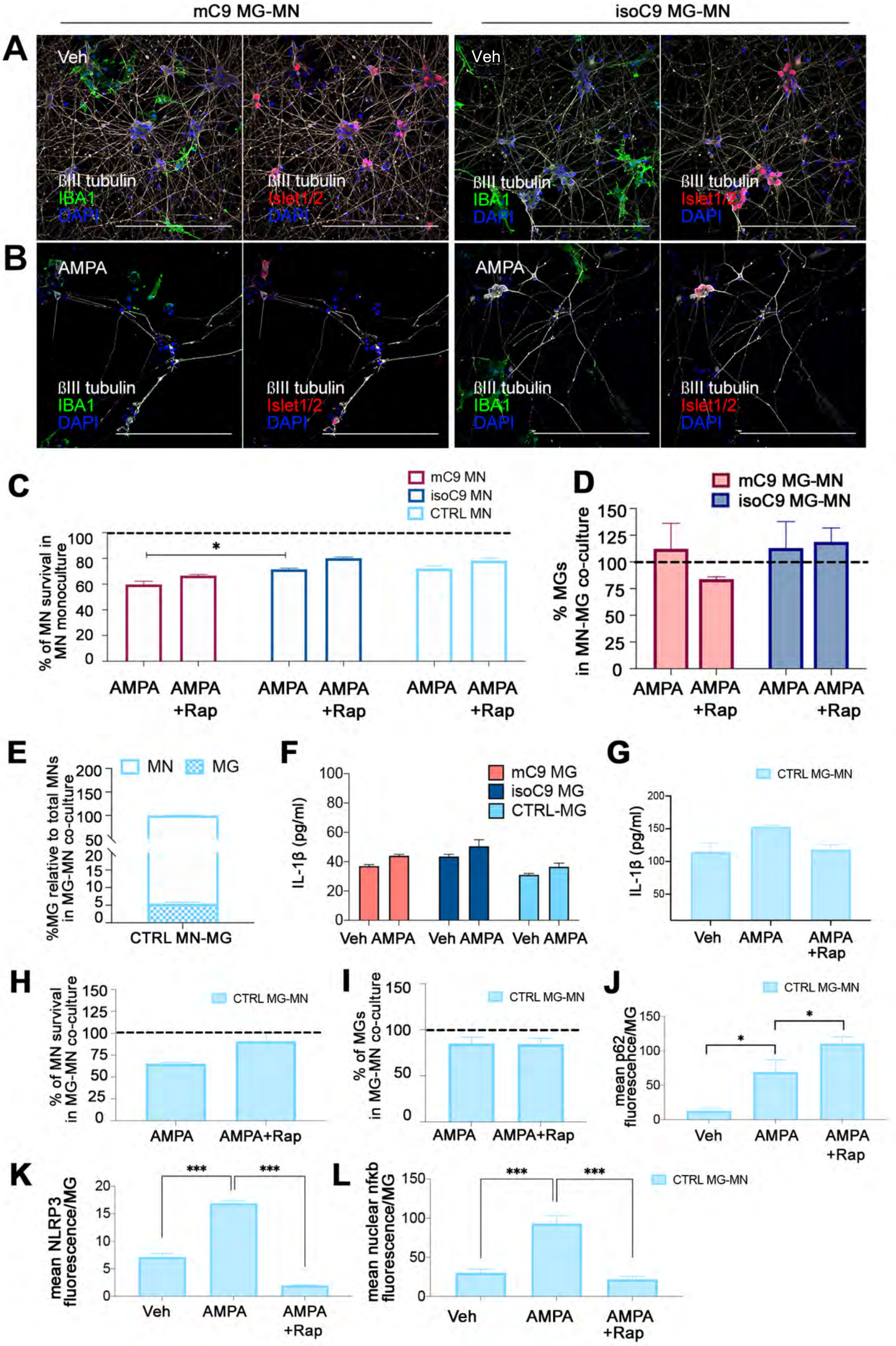

Supplementary Fig 15: Characterisation of MG-MN co-culture and assessment of neuronal death, microglial cytokine release, autophagy initiation, NF-κb signalling and NLRP3 activation following AMPA stimulation in MG-MN co-culture (A) Representative unmerged co-stained

immunofluorescence images for IBA1(green)/Islet1/2(red)/βIII tubulin (grey) in vehicle treated MG-MN co-culture (**B**) Representative unmerged co-stained immunofluorescence images for IBA1(green)/Islet1/2(red) and βIII tubulin (grey) in AMPA treated MG-MN co-culture. **(C)** Graph showing percentage of MN survival across mono-cultures of mC9-MN, isoC9-MN and CTRL-MN following AMPA treatment in absence and presence of rapamycin, vehicle treated condition is represented by the dashed line; data are represented as mean +/- SD N=3 **(D)** Graph representing the percentage survival of MGs in MG-MN co-culture relative to their vehicle treated condition (represented by the dashed line) following AMPA treatment and in presence of rapamycin. Data are represented as mean +/- SD N=3 **(E)** Graph representing the percentage of MG relative to total number of motor neurons (MN) in CTRL MG-MN co-culture. Data are represented as mean +/- SD N=3 **(F)** Graph representing IL-1β release from monocultures of mC9-MG, isoC9-MG and CTRL-MG following AMPA challenge. Data are represented as mean +/- SD N=3 **(G)** Graph representing the production of IL1β in CTRL MG-MN co-cultures across vehicle treated, AMPA treated and rapamycin treated condition. Data are represented as mean +/- SD N=3 **(H)** Graphs showing the percentage of motor neuron survival in CTRL MG-MN co-culture following AMPA treatment for 24 h in absence and presence of rapamycin relative to their vehicle treated controls as represented by the dashed line **(I)** Graph representing the proportion of MG in CTRL MG-MN co-culture relative to their vehicle treated condition (represented by the dashed line) following AMPA treatment in absence and presence of rapamycin. Data are represented as mean +/- SD N=3 **(J,K,L)** Graph representing the quantification of the mean fluorescence intensity of p62/NLRP3/NFκb per microglial cell in CTRL MG-MN co-culture across vehicle treated, AMPA treated conditions in absence and presence of rapamycin. 15 microglial cells from three biological replicates have been analysed per condition, data are represented as mean +/- SD N=3; N number for all experiments represents the number of times experiments were performed using cells generated from independent differentiations from iPSCs.

Supplementary Fig 16 (Suppl. To Fig 8)

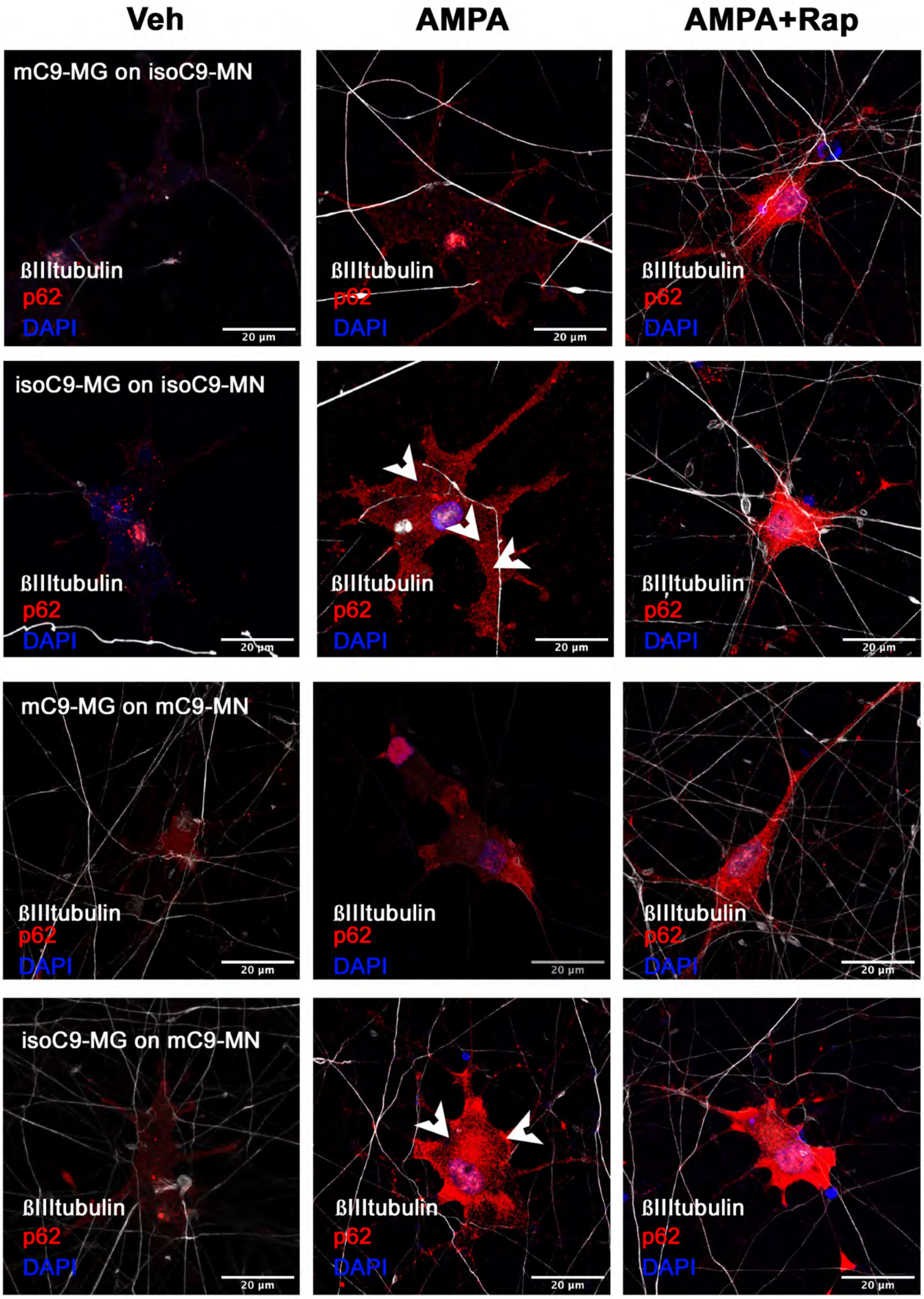

Supplementary Fig 16: p62 staining in mis-matched genotype MG-MN co-culture: Representative images of immunofluorescence staining for microglial p62 across vehicle treated, AMPA treated conditions in genotype matched MG-MN co-cultures such as -mC9MG:mC9MN, isoC9MG:isoC9MN and genotype mismatched MG-MN co-cultures - mC9MG:isoC9MN,

isoC9MG:mC9MN, isoC9 MG-MN co-culture. For quantification of p62+ve puncta across all combinations, please see main Fig 8G.

Supplementary Fig 17 (Suppl. To Fig 9)

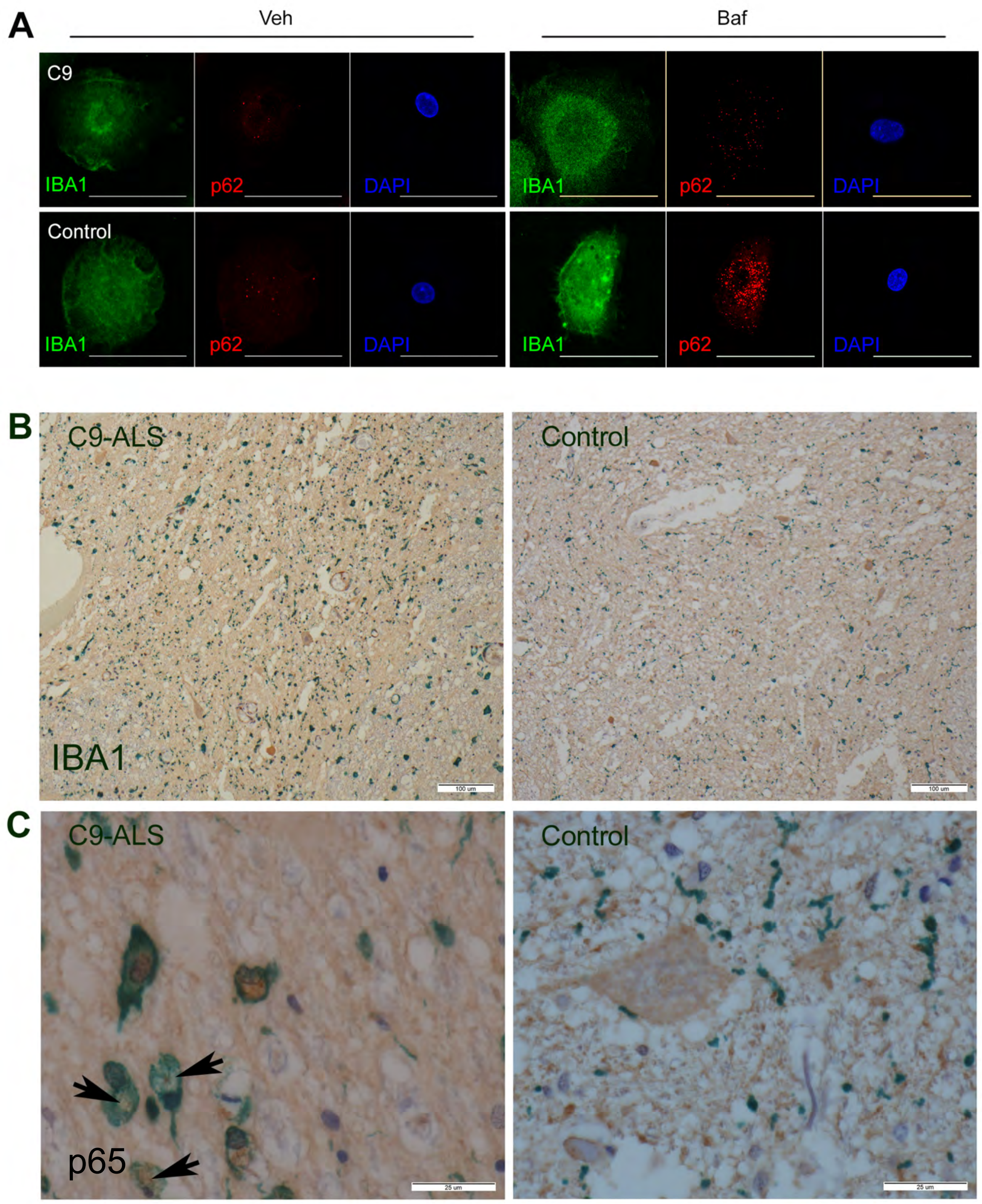

Supplementary Fig 17: Validation of disrupted autophagy and increased activation of NF-κb pathway in blood-derived macrophages and C9-ALS spinal cord samples(A) Representative co-stained immunofluorescence images for IBA1 and p62 across C9-ALS and their age and sex-matched

control showing reduced accumulation of p62 positive puncta in C9 blood macrophages compared to controls following bafilomycin treatment for 6 h. **(B)** Lower magnification images for IBA1and p65 immunostaining in the spinal cord of C9-ALS case and age- and sex-matched control showing increased microgliosis in C9-ALS cases. Scale bar=100 µm. **(C)** Higher magnification of IBA1 and p65 co-immunostaining in spinal cord of C9-ALS case and age- and sex-matched control showing increased localisation of p65 (indicated by black arrow-heads) in IBA1 positive microglial cells in C9-ALS cases when compared to age- and sex-matched control.

Supplementary Table: 1 Details of human iPSC *C9orf72* lines and control lines

**Line**	**Reprogra-mming method**	**Repeat Length**	**Sex**	**Ethnicity**	**Age of onset (years)**	**Age at skin biopsy (years)**	**Disease duration (months)**	**Diagnosis**	**Site of onset**	**Dementia**
mC9-1	Sendai	750	F	Dutch	38	39	31	ALS/ FTD	Behavioural change, language loss, wasting of hand muscles	Yes
mC9-2	Retroviral	960	M	Dutch	65	67	36	ALS	Lower limb	No
mC9-3	Reroviral	638	M	Caucasian	52	58	72	ALS	Lower limb	Cognitive decline
CTRL-1	Episomal		F	Caucasian	56					
CTRL-2	Retroviral		F	Unkmown	40					

Supplementary Table: 2 Raw data for C9ORF72 interactome (attached as a separate xcel file)

Supplementary Table 3: Sequences of fragments constituting the EGFP targeting vector. HA = homology arm. EGFP = enhanced green fluorescent protein.

Fragment	Sequence (5'→3')
Left HA	TAAAGTATTTCTGTTGTTAGGTGTTGTATTACTTTTCTAAGATTACT<br>TAACAAAGCACCACAAACTGAGTGGCTTTAAACAACAGCAATTTA<br>TTCTCTCACAATTCTAGAAGCTAGAAGTCCGAAATCAAAGTGTTGA<br>CAGGGGCATGATCTTCAAGAGAGAAGACTCTTTCCTTGCCTCTTCC<br>TGGCTTCTGGTGGTTACCAGCAATCCTGAGTGTTCCTTTCTTGCCTT<br>GTAGTTTCAACAATCCAGTATCTGCCTTTTGTCTTCACATGGCTGTC<br>TACCATTTGTCTCTGTGTCTCCAAATCTCTCTCCTTATAAACACAGC<br>AGTTATTGGATTAGGCCCCACTCTAATCCAGTATGACCCCATTTTA<br>ACATGATTACACTTATTTCTAGATAAGGTCACATTCACGTACACCA<br>AGGGTTAGGAATTGAACATATCTTTTTGGGGGACACAATTCAACCC<br>ACAAGTGTCAGTCTCTAGCTGAGCCTTTCCCTTCCTGTTTTTCTCCT<br>TTTTAGTTGCTATGGGTTAGGGGCCAAATCTCCAGTCATACTAGAA<br>TTGCACATGGACTGGATATTTGGGAATACTGCGGGTCTATTCTATG<br>AGCTTTAGTATGTAACATTTAATATCAGTGTAAAGAAGCCCTTTTT<br>TAAGTTATTTCTTTGAATTTCTAAATGTATGCCCTGAATATAAGTA<br>ACAAGTTACCATGTCTTGTAAAATGATCATATCAACAAACATTTAA<br>TGTGCACCTACTGTGCTAGTTGAATGTCTTTATCCTGATAGGAGAT<br>AACAGGATTCCACATCTTTGACTTAAGAGGACAAACCAAATATGT<br>CTAAATCATTTGGGGTTTTGATGGATATCTTTAAATTGCTGAACCT<br>AATCATTGGTTTCATATGTCATTGTTTAGATATCTCCGGAGCATTTG<br>GATAATGTGACAGTTGGAATGCAGTG
EGFP	ATGGTGAGCAAGGGCGAGGAGCTGTTCACCGGGGTGGTGCCCATC<br>CTGGTCGAGCTGGACGGCGACGTAAACGGCCACAAGTTCAGCGTG<br>TCCGGCGAGGGCGAGGGCGATGCCACCTACGGCAAGCTGACCCTG<br>AAGTTCATCTGCACCACCGGCAAGCTGCCCGTGCCCTGGCCCACCC<br>TCGTGACCACCCTGACCTACGGCGTGCAGTGCTTCAGCCGCTACCC<br>CGACCACATGAAGCAGCACGACTTCTTCAAGTCCGCCATGCCCGA<br>AGGCTACGTCCAGGAGCGCACCATCTTCTTCAAGGACGACGGCAA<br>CTACAAGACCCGCGCCGAGGTGAAGTTCGAGGGCGACACCCTGGT<br>GAACCGCATCGAGCTGAAGGGCATCGACTTCAAGGAGGACGGCAA<br>CATCCTGGGGCACAAGCTGGAGTACAACTACAACAGCCACAACGT<br>CTATATCATGGCCGACAAGCAGAAGAACGGCATCAAGGTGAACTT<br>CAAGATCCGCCACAACATCGAGGACGGCAGCGTGCAGCTCGCCGA<br>CCACTACCAGCAGAACACCCCCATCGGCGACGGCCCCGTGCTGCT<br>GCCCGACAACCACTACCTGAGCACCCAGTCCGCCCTGAGCAAAGA<br>CCCCAACGAGAAGCGCGATCACATGGTCCTGCTGGAGTTCGTGAC<br>CGCCGCCGGGATCACTCTCGGCATGGACGAGCTGTACAAG
Right HA	ATGTCGACTCTTTGTCCACCGCCATCTCCAGCTGTTGCCAAGACAG<br>AGATTGCTTTAAGTGGCAAATCACCTTTATTAGCAGCTACTTTTGC<br>TTACTGGGACAATATTCTTGGTCCTAGAGTAAGGCACATTTGGGCT<br>CCAAAGACAGAACAGGTACTTCTCAGTGATGGAGAAATAACTTTT<br>CTTGCCAACCACACTCTAAATGGAGAAATCCTTCGAAATGCAGAG<br>AGTGGTGCTATAGATGTAAAGTTTTTTGTCTTGTCTGAAAAGGGAG<br>TGATTATTGTTTCATTAATCTTTGATGGAAACTGGAATGGGGATCG

	CAGCACATATGGACTATCAATTATACTTCCACAGACAGAACTTAGT TTCTACCTCCCACTTCATAGAGTGTGTGTTGATAGATTAACACATA TAATCCGGAAAGGAAGAATATGGATGCATAAGGTAAGTGATTTTT CAGCTTATTAATCATGTTAACCTATCTGTTGAAAGCTTATTTTCTGG TACATATAAATCTTATTTTTTTAATTATATGCAGTGAACATCAAAC AATAAATGTTATTTATTTTGCATTTACCCTATTAGATACAAATACA TCTGGTCTGATACCTGTCATCTTCATATTAACTGTGGAAGGTACGA AATGGTAGCTCCACATTATAGATGAAAAGCTAAAGCTTAGACAAA TAAAGAAACTTTTAGACCCTGGATTCTTCTTGGGAGCCTTTGACTC TAATACCTTTTGTTTCCCTTTCATTGCACAATTCTGTCTTTTGCTTAC TACTATGTGTAAGTATAACAGTTCAAAGTAATAGTTTCATAAGCTG TTGGTCATGTAGCCTTTGGTCTCTTTAACCTCTTTGCCAAGTTCCCA GGTTCATAAAATGAGGAGGTTGAATGGAATGGTTCCCAAGAGAAT TCCTTTTAATCTTACAGAAATTATTGTTTTCCTAAATCCTGTAGTTG AATATATAATGCTATTTACATTTCAGTATAGTTT

Supplementary Table 4: Resource Table

PRODUCT	CATALOGUE NUMBER	COMPANY	WORKING CONCENTRATIO N
**Immunohistochemistry: primary and secondary antibodies**			
Rabbit anti-TMEM119 (human, C terminus)	ab185333	Abcam	1:200
Rabbit anti-PU.1/Spi1	ab76543	Abcam	1:200
Rabbit anti-P2Y12 (human, extracellular domain)	ab188968	Abcam	1:200
Goat anti-IBA-1	ab5076	Abcam	1:500
Rabbit anti-NF-κB p65	ab16502	Abcam	1:300
Rabbit P62/SQSTM1	18420-1-AP	Proteintech	1:700
Mouse LC3	ALX-803-082-C100	Enzo lifesciences	1:300
Donkey anti-Goat IgG (H+L) Cross-Adsorbed, Alexa Fluor® 488	A-11055	Thermo Fisher Scientific	1:1,000
Donkey anti-Rabbit IgG (H+L) Highly Cross-Adsorbed, Alexa Fluor® 647	A-31573	Thermo Fisher Scientific	1:1,000
4′,6-diamidino-2-phenylindole (DAPI)	D1306	Thermo Fisher Scientific	1:10000
**Immunoblotting: primary and secondary antibodies**			
Rabbit anti-C9orf72 S-14	sc-138763	Santacruz	1:2000
Rabbit P62/SQSTM1	18420-1-AP	Proteintech	1:2000
Rb LC3	NB600-1384	Novus biologicals	1:2000
Rb NLRP3	mAb #15101	Cell Signaling Technology	1:700
Rb GFP	2956S	Cell Signalling	
Ms GAPDH	CB100-500UG	Millipore	1:10,000
Anti-Rb HRP	1706515	Bio Rad	1:10,000
Anti-ms HRP	P044701	Agilent Technologies	1:10,000
Anti-goat HRP	Ab97110	Abcam	1:10,000

10 to 20%, Tris-Glycine	XP10205BOX	Thermo Fisher Scientific	NA
**Co-IP Experiments**			
GFP-Trap® beads	gtma-10	Chromotek	10ul
HEPES	H3375- 500g	Sigma	20mM
NaCl	71376-1kg	Sigma	150mM
MgCl2	M2670-100g	Sigma	5mM
Glycerol	49767-100ml	Sigma	10%
NP40			0.5%
sodium glycerophosphate	50020-100g	Sigma	10mM
sodium pyrophosphate	S8010	Sigma	10mM
microcystin-LR			0.1uM
sodium orthovanadate	S6508	Sigma	1mM
GTPgS	G8634	Sigma	100nM
EDTA-free protease inhibitor cocktail	469315901	Merck	
**IHC on Post-mortem tissue**			
IBA-1	ab178846	Abcam	1:3000
**Cell culture**			
Essential 8™ Medium	A1517001	Gibco	NA
Mouse (ICR) Inactivated Embryonic Fibroblasts	A24903	Gibco	NA
Collagenase Type IV	17104019	Thermo Fisher Scientific	1mg/ml
DispaseII	17105041	Thermo Fisher Scientific	0.5mg/ml
Rock Inhibitor	1254	Tocris	10μM
X-VIVO 15 Serum-free Hematopoietic Cell Medium	BE02-060F	Lonza	NA
Advanced DMEM/F12	12634028	Thermo Fisher Scientific	0.5X
Neurobasal	21103049	Thermo Fisher Scientific	0.5X
GlutaMAX™ Supplement	35050061	Thermo Fisher Scientific	1X
MEM Non-Essential Amino Acids Solution (100X)	11140050	Thermo Fisher Scientific	1X

N-2 Supplement (100X)	17502048	Thermo Fisher Scientific	1X
β-Mercaptoethanol	31350-010	Thermofisher Scientific	100µM
Antibiotic Antimycotic Solution (100×)	A5955	Merck	1X
RPMI 1640 Medium with L-Glutamine	12-702Q	Lonza	NA
Human MCSF	300-25	Peprotech	100ng/ml
Human IL 3	PHC0035	Gibco	25ng/ml
Human GMCSF	300-03	Peprotech	10ng/ml
Human IL34	200-34	Peprotech	100ng/ml
Human BMP4	120-05ET	Peprotech	50ng/ml
Human VEGF	100-20	Peprotech	50ng/ml
Human SCF	300-07	Peprotech	25ng/ml
bafilomycin	1334-10	Tocris	100nM
rapamycin	9904	Cell Signalling	10µM
Dimethyl sulfoxide (DMSO)	D2650	Merck	NA
LPS	L4391	Merck	100ng/ml
pHrodo™ Red Zymosan Bioparticles™ Conjugate for Phagocytosis	P35364	Thermo Fisher Scientific	
Poly-D-lysine hydrobromide	P7280	Merck	0.01%
Poly-L-ornithine	P4957	Sigma	100µg/ml
Gelatin	G1393	Merck	0.1%
DPBS	14040133	Invitrogen	NA
EBSS	14155-048	Gibco	
7.5% BSA	A8412	Sigma	0.1%
D-(+) Glucose 45%	G8769	Sigma	0.45%
FBS	11550356	Thermo Fisher Scientific	2.5%
1M $MgCl_2$	M2670	Sigma	2mM
0.5M EDTA pH8.0	15575-038	Invitrogen	0.8mM
Accutase	A6964	Merck	1X
B27 with Vit A	17504044	Thermo Fisher Scientific	1X
L-Ascorbic acid	A4403	Sigma-Aldrich	2.5µMMN-NF & 10µM MN diff Base
Ascorbic acid	A4403	Sigma-Aldrich	2.5 µM
Retinoic acid	R2625	Sigma-Aldrich	0.1µM MN diff base

			&1µM MN-NF
Smoothened agonist (SAG)	566660	Sigma-Aldrich	0.5 µM
BDNF	248-BDB	R & D Systems	10 ng/ml
GDNF	212-GD/CF	R & D Systems	10 ng/ml
DAPT	2634	Tocris Bio-Techne	10 µM
CNTF	257-NT/CF	R & D Systems	10ng/ml
IGF-1	AF-100–11	PeproTech	10ng/ml
FGF-2	450-33	PeproTech	10ng/ml
uridine	U3003	Sigma-Aldrich	1µM (1:10,000)
5-Fluoro-2-deoxyuridine	F0503	Sigma -Aldrich	25µM (1:1000)
DNAse	DN25	Sigma-Aldrich	0.05mg/ml
0.25% Trypsin–EDTA	25200-056	Thermo Fisher Scientific	0.05%
L-Glutamic acid	G5889	Sigma-Aldrich	25mM
Laminin	L2020	Sigma-Aldrich	5.0 µg/ml
Fibronectin	F2006	Sigma-Aldrich	10 µg/ml for and 20µg/ml for glass coverslip
Matrigel Growth factor Reduced	354230	Corning	1:30 for IPSC, 1:20 for MNs
Adenosine 5′-triphosphate disodium salt hydrate	A7699	Sigma-Aldrich	1mM
**Flow Cytometry**			
APC anti-CD45 (clone 2D1)	368511	Biolegend	1:250
APC anti-CD11b (clone ICRF44)	301309	Biolegend	1:300
**Miscellaneous**			
Chemiluminescence kit	12316992	Fisher	NA
TritonX100	X100	Merck	0.03%
BSA	EQBAC62	Europa Bioproducts	3%
Para-formaldehyde	AGR1026	Elektron microscopy	4%
FluorSave	345789	Merk	NA
BCA Protein Assay kit	23227	Pierce	NA
Hyperfilm	28-9068-37	Amersham	NA
Human IL-6 duo set ELISA	DY206-05	R&D Systems	NA
Human IL-1beta duo set ELISA	DY201-05	R&D Systems	NA
BD Vacutainer® CPT™	362782	BD Biosciences	NA
40micron cell strainers	08-771	Fisher Scientific	

PVDF Transfer Membrane, 0.2 µm	88520	Thermo Fisher Scientific	
ROCHE cOmplete™ Protease Inhibitor Cocktail	12352200	Millipore Sigma	
9cm^2 petri dishes	PDS-149-050F	Thermo Fisher Scientific	
500ml Rapid Flow unit	10199-655	Thermo Fisher Scientific	
**Primer Name**	**Sequence (5'➔3')**		
T7_Fwd	ACAGGATTCCACATCTTTGACT		
T7_Rev	GCGATCCCCATTCCAGTTTC		
5'HA_Fwd	CGGCGATATCGGATCCATATGACGTTAAAGTATTTCTGTTGTTAGGTG		
5'HA_Rev	TCACCATGGTGGCCACTGCATTCCAACTGTC		
EGFP_Fwd	TTGGAATGCAGTGGCCACCATGGTGAGCAAG		
EGFP_Rev	AAAGAGTCGACATCTTGTACAGCTCGTCCATG		
3'HA_Fwd	CGAGCTGTACAAGATGTCGACTCTTTGCCCAC		
3'HA_Rev	ATGATTACGCCAAGCTCGCGAGGCCAAACTATACTGAAATGTAAATAGCATTATATATTCAAC		
C9Ext_Fwd	TGGGTTCTGTCTTGGATGTG		
C9_Rev	GCGATCCCCATTCCAGTTTC		
